# The association of rs17713054 with Neanderthal origin at 3p21.31 locus with the severity of COVID-19 in Iranian patients

**DOI:** 10.1038/s41598-024-65732-8

**Published:** 2024-07-01

**Authors:** Mohammad Yaghmouri, Javad Safdari Lord, Masoumeh Amini, Mir Saeed Yekaninejad, Pantea Izadi

**Affiliations:** 1 Department of Medical Genetics, School of Medicine, Tehran University of Medical Sciences, Tehran, Iran; 2 Department of Epidemiology and Biostatistics, School of Public Health, Tehran University of Medical Sciences, Tehran, Iran

**Keywords:** Evolution, Genetics, Microbiology, Molecular biology, Diseases, Medical research, Molecular medicine, Pathogenesis, Risk factors

## Abstract

Since the COVID-19 pandemic, the diversity of clinical manifestations in patients has been a tremendous challenge. It seems that genetic variations, as one of the players, contribute to the variety of symptoms. Genome-wide association studies have demonstrated the influence of certain genomic regions on the disease prognosis. Particularly, a haplotype at 3p21.31 locus, inherited from Neanderthals, showed an association with COVID-19 severity. Despite several studies regarding this haplotype, some key variants are not sufficiently addressed. In the present study, we investigated the association of rs17713054 at 3p21.31 with COVID-19 severity. We analyzed the genotype of 251 Iranian COVID-19 patients (151 patients with asymptomatic to mild form as control and 100 patients with severe to critical symptoms without any comorbidities as case group) using the ARMS-PCR method. Results demonstrated that the A allele confers an almost twofold increased risk for COVID-19 severity (*P* *value* = 0.008). The AA genotype also raises the risk by more than 11 times following the recessive model *(P* *value* = 0.013). In conclusion, the A allele in rs17713054 was a risk allele in Iranian patients and was independently associated with COVID-19 severity. More studies are beneficial to confirm these findings in other populations and to develop strategies for risk assessment, prevention, and personalized medicine.

## Introduction

Since the COVID-19 pandemic, the world has faced a wide variety of impediments, one of which is the wide range of clinical manifestations that have surfaced. Patients display a range of symptoms, from asymptomatic to catastrophic^1,2^. The majority of patients, however, show non-severe symptoms. To explain this disparity, several risk factors were addressed, with age (≥ 65 years), male gender, and comorbidities (such as diabetes, hypertension, chronic respiratory diseases, chemotherapy, etc.) being among the three major factors adversely affecting the COVID-19 severity^3^. Nonetheless, the risk factors described above do not apply to all patients with severe disease; therefore, it remains a challenging intricacy that must be addressed properly. Genetic predisposition may play a pivotal role which could explain this wide spectrum of symptoms. Several Genome-wide association studies (GWAS) were conducted to identify genes and variants contributing to COVID-19 severity^4–6^. Among several loci detected in GWAS, the 3p21.31 locus has shown a substantial association with COVID-19 severity^7^. Moreover, previous studies have demonstrated the role of this locus in coronary artery disease, type 1 diabetes, and rheumatoid arthritis as a disease with dysregulated immune responses^8–10^. One of the key variants in this locus which was considered to affect the outcome of COVID-19 is rs17713054 G > A. This polymorphism resides in an enhancer region at the 3p21.31 locus. Also, it is part of a novel gene^11^. This novel gene with the ensemble ID ENSG00000288720 is a lncRNA that codes three RNA transcripts and, to the best of our knowledge, its function is unknown. The A allele of the rs17713054 variant generates a motif for a group of transcription factors called C/EBPβ. Thus, it leads to the interaction of the enhancer with the promoter of the LZTFL1 gene at the 3p21.31 locus, consequently, upregulation of LZTFL1 occurs and, the epithelial-mesenchymal transition (EMT) process is suppressed which suggests exacerbating the COVID-19 outcome^11^. Interestingly, this variant is a member of the haplotype at the 3p21.31 locus that is inherited from Neanderthals^12^. Additionally, the role of introgressed segments of the Neanderthal genome in present-day human cancer and expression regulation was indicated^13,14^. The discovery that different segments of the Neanderthal genome were introgressed into the human genome was due to the hard work of Svante Pääbo. et al.^12,15,16^. Their discoveries were so significant that Svante Pääbo was awarded the 2022 Nobel Prize^17^. In 2020, a survey study demonstrated that rs17713054 is associated with hospitalization in patients of European ancestry^18^. Another investigation in Russian patients showed an association between rs17713054 and COVID-19 severity^19^. Additionally, another investigation of South Asian population suggested that this core haplotype was not related to severe COVID-19^20^. For clarifying the observed controversies, the present study aimed to investigate the association of rs17713054 with the severity of COVID-19 in the absence of known risk factors for severe COVID in Iranian patients.

## Materials and methods

### Study population

Two hundred and fifty-one adult COVID-19 patients voluntarily participated in this study. For SARS-CoV-2 infection diagnosis, nasopharyngeal swab samples were obtained from patients from September to December 2020 at Farmanfarmayan Health Center (Tehran, Iran). The positive COVID-19 result of the PCR test was the first criterion for participation in this study. Patients without any symptoms or mild symptoms were classified as the control group(n = 151). The case group included 100 patients with severe to critical symptoms (such as a respiratory rate of more than 30 beats per minute, oxygen saturation level (SPO2) less than 90%, lung infiltration of more than 50%, and organ failure who needed hospitalization). All patients with background diseases or other known risk factors (such as age ≥ 65 years, diabetes, hypertension, heart failure, stroke, cancer chemotherapy or immunodeficiency, etc.), were excluded from the case group.

All of the participants signed written informed consent, and then 5 ml of peripheral blood was driven from the patients in tubes with EDTA anticoagulant. This study has been approved by the ethics committee of the School of Medicine of Tehran University of Medical Sciences (Ethics code: IR. TUMS. MEDICINE.REC.1401.366) and has been performed under the Declaration of Helsinki. All experiments and techniques have been performed following relevant guidelines and regulations.

### DNA extraction

We used a salting-out technique that has been described previously to extract genomic DNA from whole blood samples^21^. Next, the quality and quantity of the extracted DNA samples were evaluated via a Nano-Drop 2000™ spectrophotometer device (Thermo Fisher Scientific, USA). Finally, these extracted genomic DNA samples were stored at – 20 °C for the genotyping step.

### Genotyping for rs17713054

**Figure 1:**
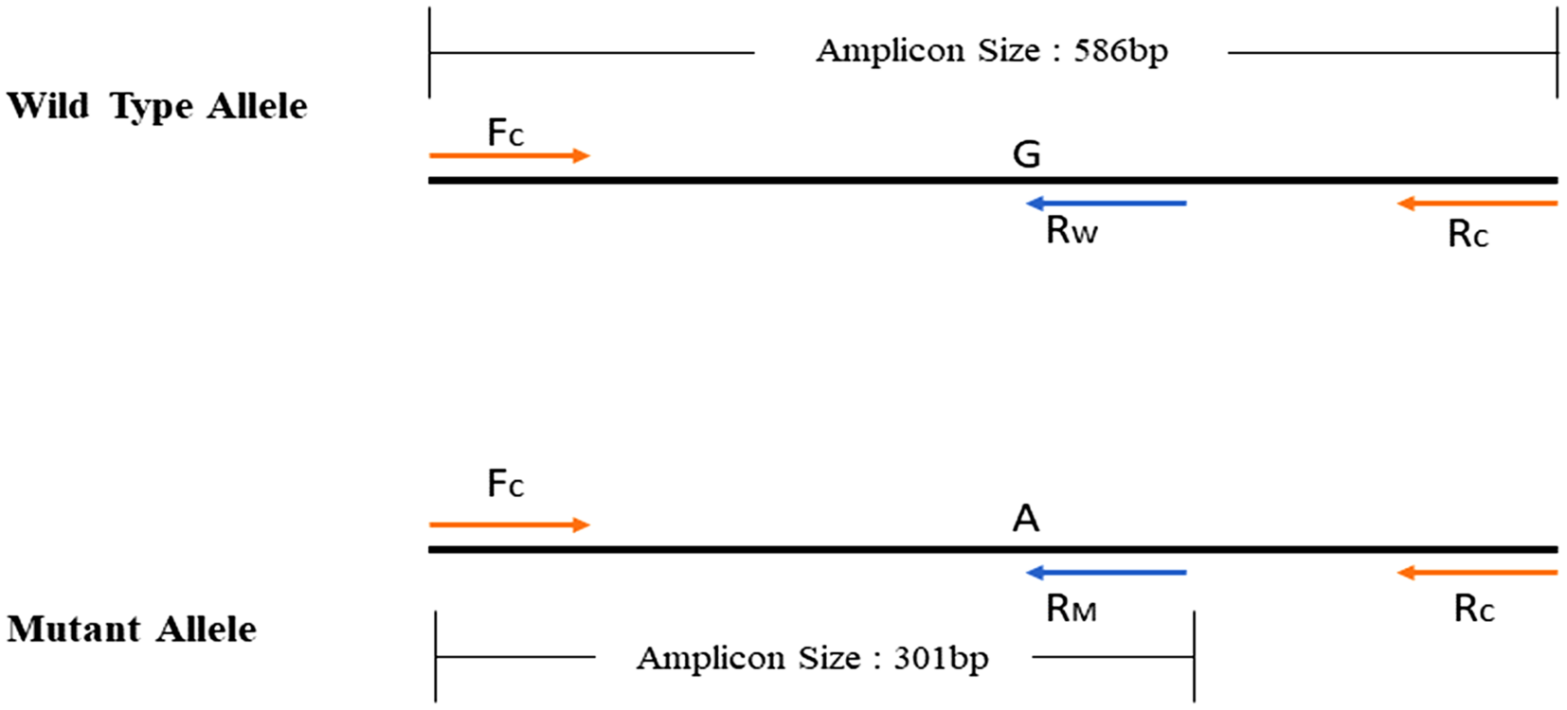
Illustration of primers’ positions for rs17713054 in the ENSG00000288720 gene. Two reactions were conducted in two tubes for each sample. In each reaction, Fc and Rc primers produced a 586 bp amplicon as an internal control, and the allele-specific primers (Rw or Rm) produced a 301 bp amplicon. Fc, Forward control primer; Rc,  Reverse control primer; Rm,  Reverse mutant primer; Rw,  Reverse wild type primer.

Using the amplification refractory mutation system polymerase chain reaction (ARMS-PCR) method, the alleles of the rs17713054 variant were detected. In this method, two separate allele-specific primers, one for the A allele and one for the G allele (Reverse mutant and Reverse wild-type, respectively), and one pair of internal control primers (Forward control and Reverse control, respectively) were designed (Table 1). The genotyping was carried out in two separate PCR reactions for each sample. One of the reaction tubes contains control primers and the reverse mutant primer (Fc, Rc, Rm) and the second one contains control primers and reverse wild-type primer (Fc, Rc, Rw). A total volume of 10ul of all reagents required for the amplification and genotyping includes 5µl of TEMPase Hot Start 2 × Master Mix A BLUE (Amplicon, Denmark), 0.5µl (5 pmol/ul) of each forward and reverse control primers respectively, 1µl (5 pmol/ul) of each of allele-specific primers, 1µl DNA sample and 2 µl of distilled water. For the blank control tube 1µl of distilled water was added instead of a DNA sample. The amplification reaction was performed in the ABI Veriti thermal cycler machine (Thermo Fisher Scientific, USA). The TOUCHDOWN PCR program was carried out. In this program, the initial annealing temperature was 61 °C and after each cycle, the temperature was decreased by 1 °C until reached 54 °C as the annealing temperature after eight cycles. After reaching the final annealing temperature, another 23 amplification cycles (30 s at 95 °C, 30 s at 54 °C, and 30 s at 72 °C) and a final extension at 72 °C for 7 min were executed to complete the PCR process. The amplification products were separated by electrophoresis on 1.5% agarose gels (SinaClon Bioscience, Iran), containing 0.5 mg/L DNA gel stain (Pishgam, Iran), and the image of the PCR products was visualized under the UV light and captured by gel documentation system (Syngene, UK). There were two amplification products of the reaction: 586 bp was the control band and must be observed in all samples to confirm that the reaction was properly performed. The 301 bp amplicon was an allele-specific band (Fig. 1).

**Table 1:** ARMS-PCR primers for rs17713054 genotyping.

Primer name	Sequences
Forward control (internal control)	5-AACAGCTCCACACCTCAGC-3′
Reverse control (internal control)	5′-GCATCTCATCCCACAGTGAAC-3′
Reverse mutant (for A allele)	5′-TCTGTTTCCTGGCCAAGTTT-3′
Reverse wild type (for G allele)	5′-TCTGTTTCCTGGCCAAGTTC-3′

### Statistical analysis

After data collection, we utilized SPSS software (version 26) to perform statistical analyses. The Chi-squared (χ2) (Fisher’s exact test) was executed to evaluate the significance of the frequency of genotypes and alleles between the case and the control groups. The association between rs17713054 and the severity of COVID-19 and the odds ratio were calculated with 95% confidence intervals (CI). The Mann–Whitney U test was performed for evaluation the relationship between gender as a known confounding factor with COVID-19 outcome. To assess whether the population study is in concert with the Hardy–Weinberg equilibrium, we used SNPanalyzer 2.0 software. Also, we investigated the model of inheritance for this polymorphism. Three different inheritance models were analyzed using SNPanalyzer 2.0. Then, to find the best fitted model for this polymorphism Akaike Information Criterion (AIC) and Bayesian Information Criterion (BIC) were assessed with SNPStats online web tool software^22^. All *P* *values* were considered statistically significant for *P* < 0.05.

## Results

A total number of 251 patients participated in this study. Patients were categorized into case and control groups and those with known risk factors such as age (> 65 years old) and pre-existing comorbidities were excluded from the study population. The case group consisted of 100 patients characterized by severe to critical COVID-19 symptoms with a mean age of 46.27 (std ± 10.18), and 151 patients were involved in the control group with no symptoms to mild symptoms and a mean age of 38.26 (std ± 11.86). The percentage of females who participated in the case and control groups was 44% and 39%, respectively. Also, gender as a confounding factor did not indicate a statistically significant difference between the case and control groups (*P* *value* = 0.364).

**Figure 2:**
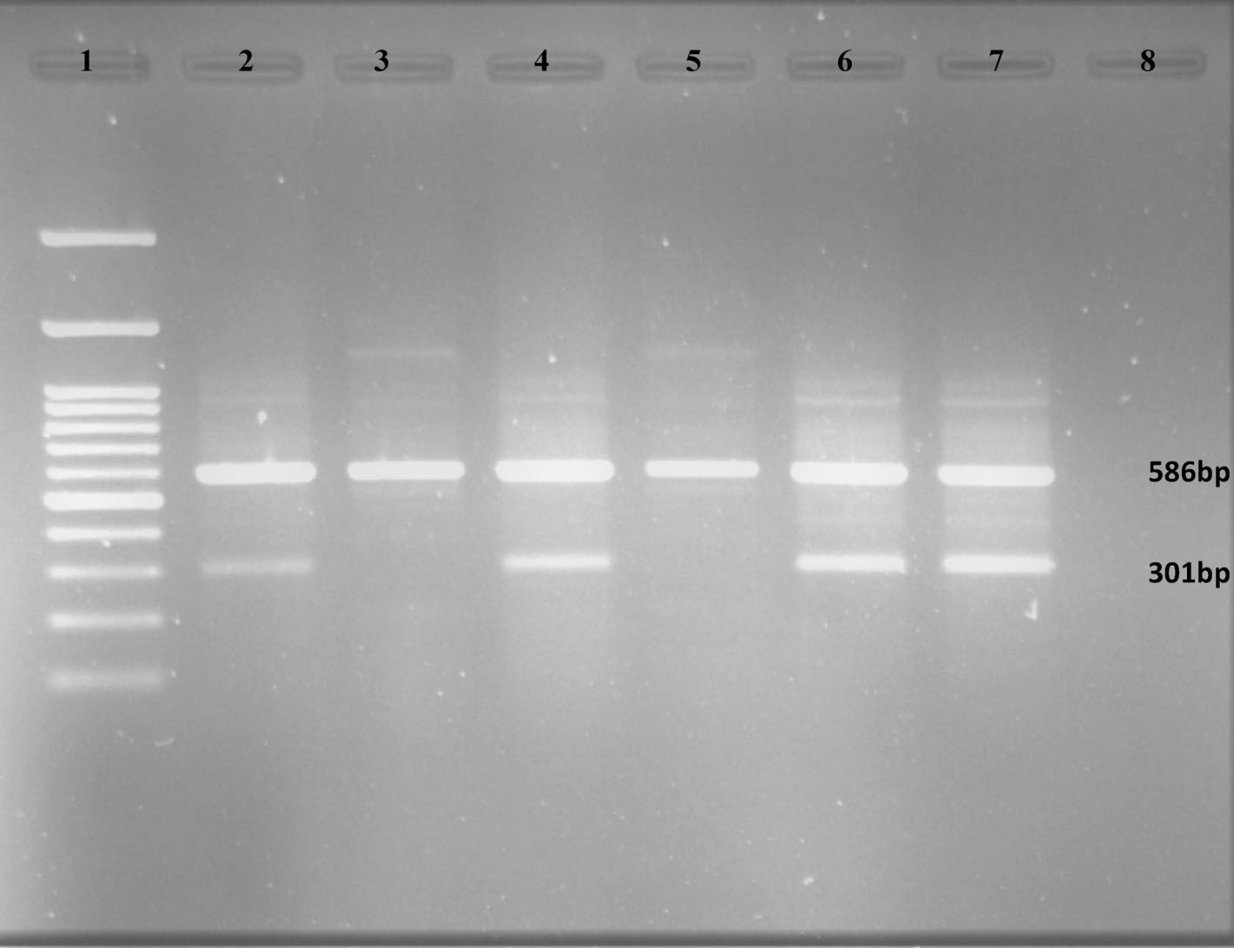
Gel electrophoresis of the amplified products showed three different genotypes for rs17713054 in three patients. To detect A and G alleles two reactions were carried out in parallele for each DNA sample. All amplified products contain a 586 bp PCR product as an internal control to check the validity of the PCR rection, while amplification of the allele-specific products with a length of 301 bp is dependent on the genotype of the each patient. Every two wells show the genotyping of a DNA sample: 1. 100 bp Ladder, 2&3. Homozygote patient with GG genotype (lanes 2 and 3 are indicative of homozygosity for the wild-type (G) allele as no product was observed for the mutant (A) allele in lane 3), 4&5. Homozygote patient with AA genotype (lanes 4 and 5 are indicative of homozygosity for the mutant (A) allele as no product was observed for the wild-type(G) allele in lane 5), 6&7. Heterozygote patients with GA genotype (lanes 6 and 7 are indicative of heterozygosity for A and G alleles as both were amplified. 8. Blank (no DNA template).

The rs17713054 variant has two alleles A/G, therefore, three genotypes (AA, GA, GG) were determined for this variant (Fig. 2). The genotype frequency and the allele frequency of the variant were detected (Table 2).

**Table 2:** Analysis of rs17713054 genotypes and alleles association with COVID-19 severity under five different inheritance models.

Genotypes/Alleles	Case group n = 100 (%)	Control group n = 151 (%)	Model of Inheritance	*P-values*	OR (95% CI)*	AIC*	BIC*
GG	70 (70%)	120 (79.5%)	–	87 × 10^–2^	0.603 (0.337–1.079)	_	
GA	24 (24%)	31 (20.5%)		2.93 × 10^–1^	1.222 (0.667–2.24)		
AA	6 (6%)	0		**8 × 10**^**–3**^	**11.2 (1.357–92.46)**		
G	164 (82%)	271 (89.7%)		**0.013**	**1.919 (1.143–3.221)**		
A	36 (18%)	31 (10.3%)					
–	–	–	Log-Additive	0.016	0.53 (0.32–0.89)	335.7	342.8
GG-AA	120(79.5%)	76(76%)	Over-Dominant	0.52	1	341.1	348.2
GA	31(20.5%)	24(24%)			0.82 (0.45–1.5)		
GG	120 (79.5)	70 (70)	Co-Dominant	0.0024	1.00	331.4	342
GA	31 (20.5)	24 (24)			0.75 (0.41–139)		
AA	0	6 (6)			0.00		
GG	120 (79.5)	70 (70)	Dominant	0.089	1.00	338.6	346
GA-AA	31 (20.5)	30 (30)			0.6 (0.34–1.08)		
GG-GA	151 (100)	94 (94)	Recessive	**0.0008**	1.00	**330.3**	**337**
AA	0	6 (6)			0.00		

*OR odds ratio, *CI* confidence intervals, *AIC* Akaike Information Criterion, *BIC* Bayesian Information Criterion.

Statistically significant values have been shown in bold.

According to the statistical analysis of the results, the distribution of 17,713,054 genotypes between the control group and the case group differed significantly (*P* *value* = 5 × 10^–3^). The frequency of the A allele increased from 10.3% in the control group to 18% in the case group and was statistically substantial, while, the frequency of the G allele slightly decreased from 89.7% in the control group to 82% in the case group and was not statistically considerable. Interestingly, no individuals were identified for the AA genotype in the control group while 6% of the case group were homozygote for the AA genotype. In contrast, For GG and GA genotypes we did not observe any considerable differences between the genotypes and the study groups (Table 2).

The population study was also evaluated for the Hardy–Weinberg equilibrium. According to the results, the frequency distribution of genotypes and alleles in the total population is under the Hardy–Weinberg equilibrium (*P value* = 0.404). The genotypes in the case and control groups also follow the Hardy–Weinberg equilibrium (*P value* = 0.0615*, P value* = 0.159, respectively).

We also assessed the model of inheritance for the alleles of rs17713054. According to the analyses Log-additive, Co-dominant, and Recessive models showed considerable differences, therefore, these models were evaluated for the Akaike information criterion (AIC) and Bayesian information criterion (BIC) using the SNPStats online tool to assess the fitness of the models. Finally, the recessive model of inheritance showed the lowest *P value* (= 0.0008), lowest AIC, and BIC, which indicates that it is the fittest model of inheritance for rs17713054 alleles (Table 2).

## Discussion

Several studies in different populations have demonstrated the link between variations in the host genome and COVID-19 outcomes^23–25^. Seemingly, it has been shown that the evolutionary background of modern humans can play a major role in the severity of the COVID-19 symptoms^12^. Recently , a haplotype at 3p21.31 related to Neanderthals has been in the spotlight, and the rs17713054 polymorphism, a member of this core haplotype, has been reported to influence COVID-19 severity^11,12^.

According to our findings, the A allele and AA genotype of rs17713054 indicated an increased risk for the severe form of COVID-19. Consistently, these findings was in concordance with results obtained from patients with European and Russian ancestry^18,19^. Interestingly, in the Russian population this risk genotype showed an odds ratio of 4.56 which was much lower compared our finding^19^. This difference might be because individuals above the age of 65 were not excluded from the mentioned study. However, such associations was not be replicated in the South Asian population^20^. It is noticble that rs17713054 was not directly investigated in the mentioned study, but instead another variant in high linkage disequilibrium with the core haplotype was selected. However, this inconsistency with the results of other investigations might be due to different genetic background of various populations.

In the present study population, the frequency of the minor allele was 13.3%. It seems that the frequency range of the A allele, as a risk variant, is from 7 to 11.5% in European populations with Italians having a frequency of 10.3%. Also, in Russian and Yakut populations an estimation of 16% and 2% was reported respectively^19,26^.In other populations like Americans and Ashkenazi groups, the frequency of the minor allele is 4–5% and 13.8%, in turn. The A allele reaches its maximum frequency (nearly 30%) in South Asian populations, in contrast, its minimum frequency (approximately 0–1%) was observed in African and East Asian populations. This means that the risk allele frequency in our study population is much closer to the European populations and this frequency is less than half of the allele frequency in South Asian populations^27,28^.

Although the present association study was in line with previous GWAS in populations of European ancestries to confirm their findings regarding the rs17713054 variant, there is evidence that this variant affects the severity of COVID-19 via a putative mechanism^11^. The rs17713054G > A variant is located in a novel gene which is a lncRNA (ensemble ID ENSG00000288720) and codes three transcripts, however, there is not enough information regarding this lncRNA which calls for more functional studies. Also, this rs17713054 resides within a region that has the characteristics of an enhancer. It has been shown that the A allele can generate a motif for C/EBPβ transcription factors and upregulate the expression of the LZTFL1 gene^11^. This gene is one of the genes that represented a considerable association with severe COVID-19 outcomes^7^. Previously, the LZTFL1 gene was known for its role as a tumor suppressor gene in cancers^29^. The LZTFL1 upregulation sequesters the EMT process and improves the survival rate in some cancers as well as inhibiting the metastatic effect of the EMT^30,31^. Additionally, this process plays a role in cell repair and immune responses^32^, thus, due to inhibition of the EMT process via the LZTFL1 upregulation, its beneficial functions are hampered. LZTFL1 gene is highly expressed in the lung ciliated epithelial cells which are critical cellular targets for SARS-COV-2^11^. However, more functional studies are required to better understand how rs17713054 influences LZTFL1 expression and EMT process in the context of COVID-19 severity.

We had some limitations in our study such as limited sample size. Further studies with larger sample sizes are warranted to validate our results. Additionally, our study focused on the Iranian population, and the genetic background may differ in other populations. Thus, caution should be exercised when generalizing these findings to other ethnic groups.

In conclusion, the findings of this study demonstrate a substantial association between the A allele of rs17713054 and the severity of COVID-19. Specifically, patients who were homozygous for the A allele exhibited an 11-fold increased risk of developing the severe form of the disease. These results shed light on the genetic factors that contribute to the variability in COVID-19 outcomes and highlight the importance of genetic risk assessment in identifying individuals at higher risk for severe illness. By identifying these genetic markers, healthcare providers can prioritize at-risk patients for intensive treatment and personalized medical interventions, ultimately aiming to reduce mortality rates.

### Supplementary Information

Supplementary Information 1.

Supplementary Information 2.

## Data Availability

All data generated or analyzed during this study are included in the supplementary information file.
