# Supplementary material for: The association of rs17713054 with Neanderthal origin at 3p21.31 locus with the severity of COVID-19 in Iranian patients
Source: Sci Rep. 2024 Jul 1;14:15058. doi: 10.1038/s41598-024-65732-8 (PMC11219939; doi:10.1038/s41598-024-65732-8)
Supplement: Supplementary file 1 — Supplementary Information 1. [file 41598_2024_65732_MOESM1_ESM.pdf]

Supplementary figure 1. Images of agarose gel electrophoresis of the amplified products for genotyping of DNA samples for rs17713054(A/G). L= Ladder, B = Blank (No DNA control), × = Inconclusive results (No products or weak bands)
** Samples with either no products or weak bands were repeated for genotyping

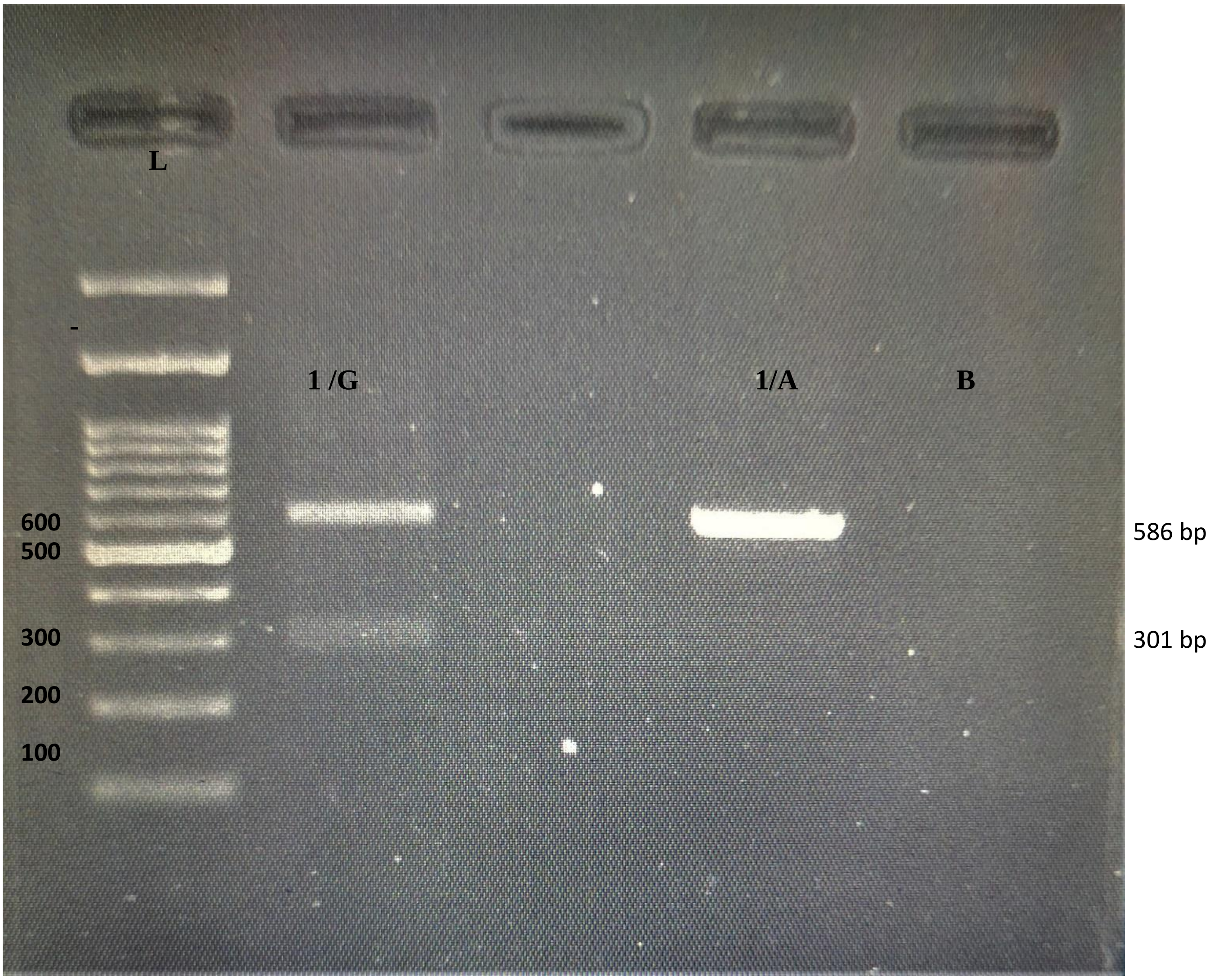

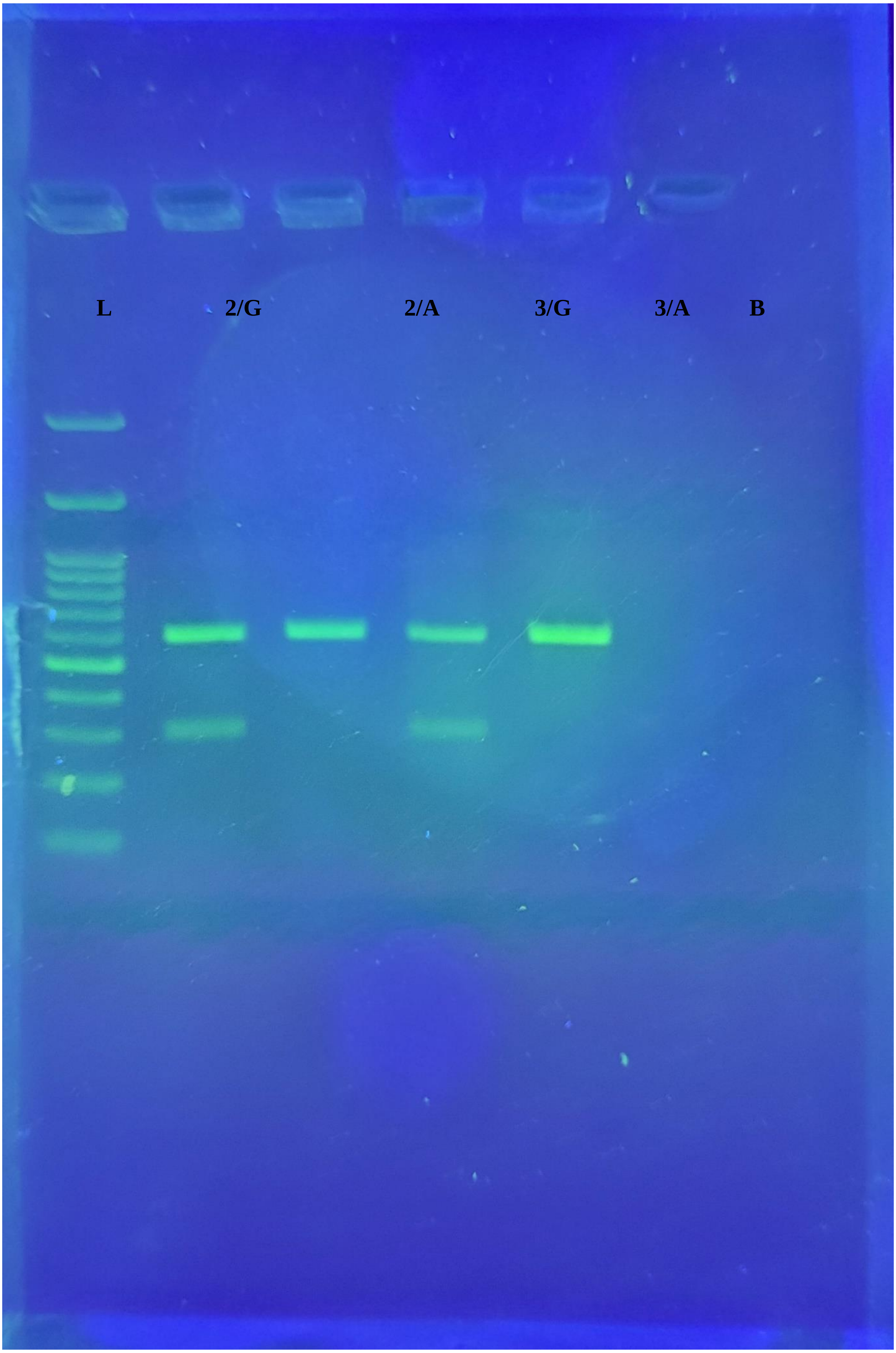
L
2/G
2/A
3/G
3/A
B

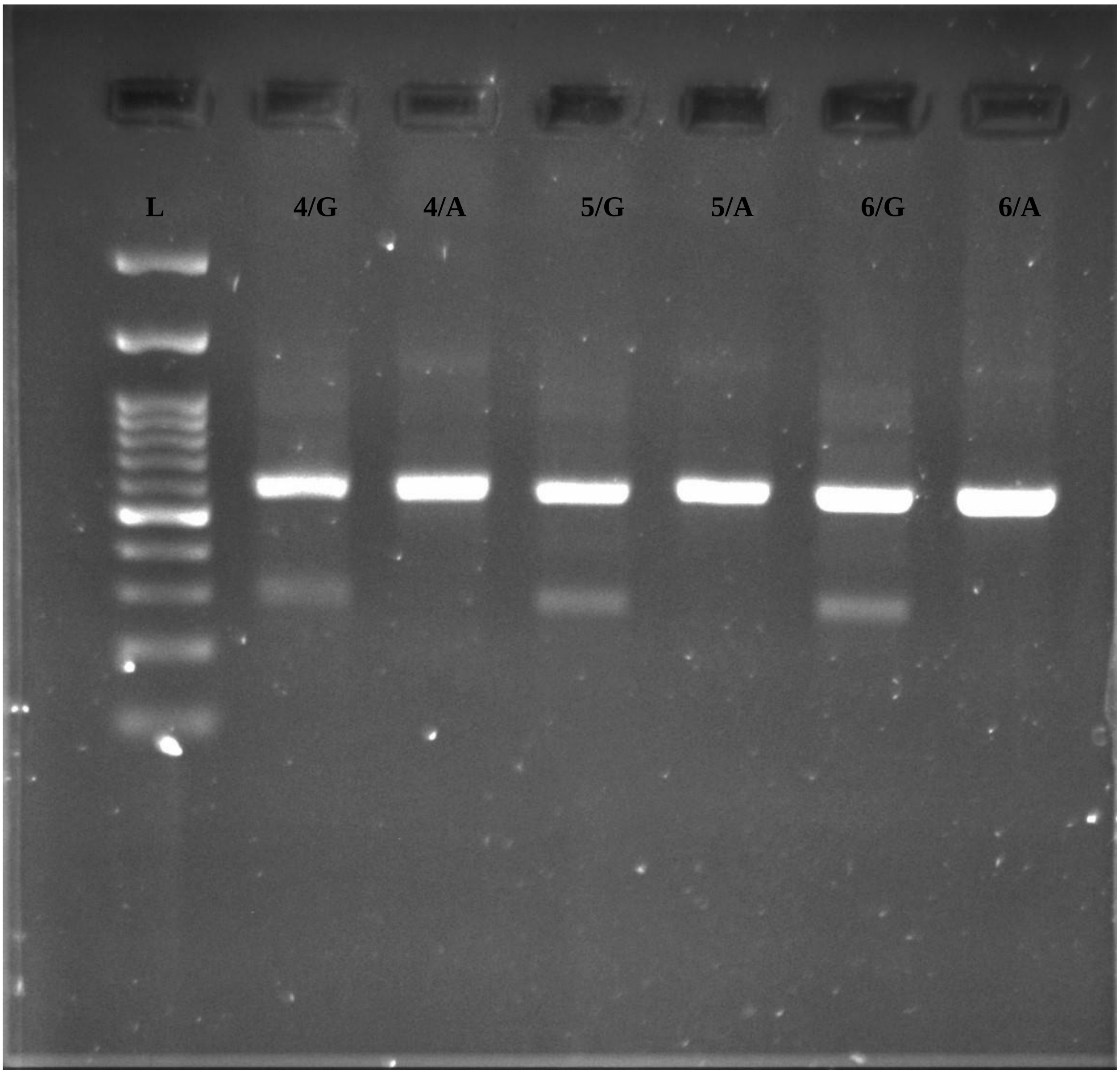
L
4/G
4/A
5/G
5/A
6/G
6/A

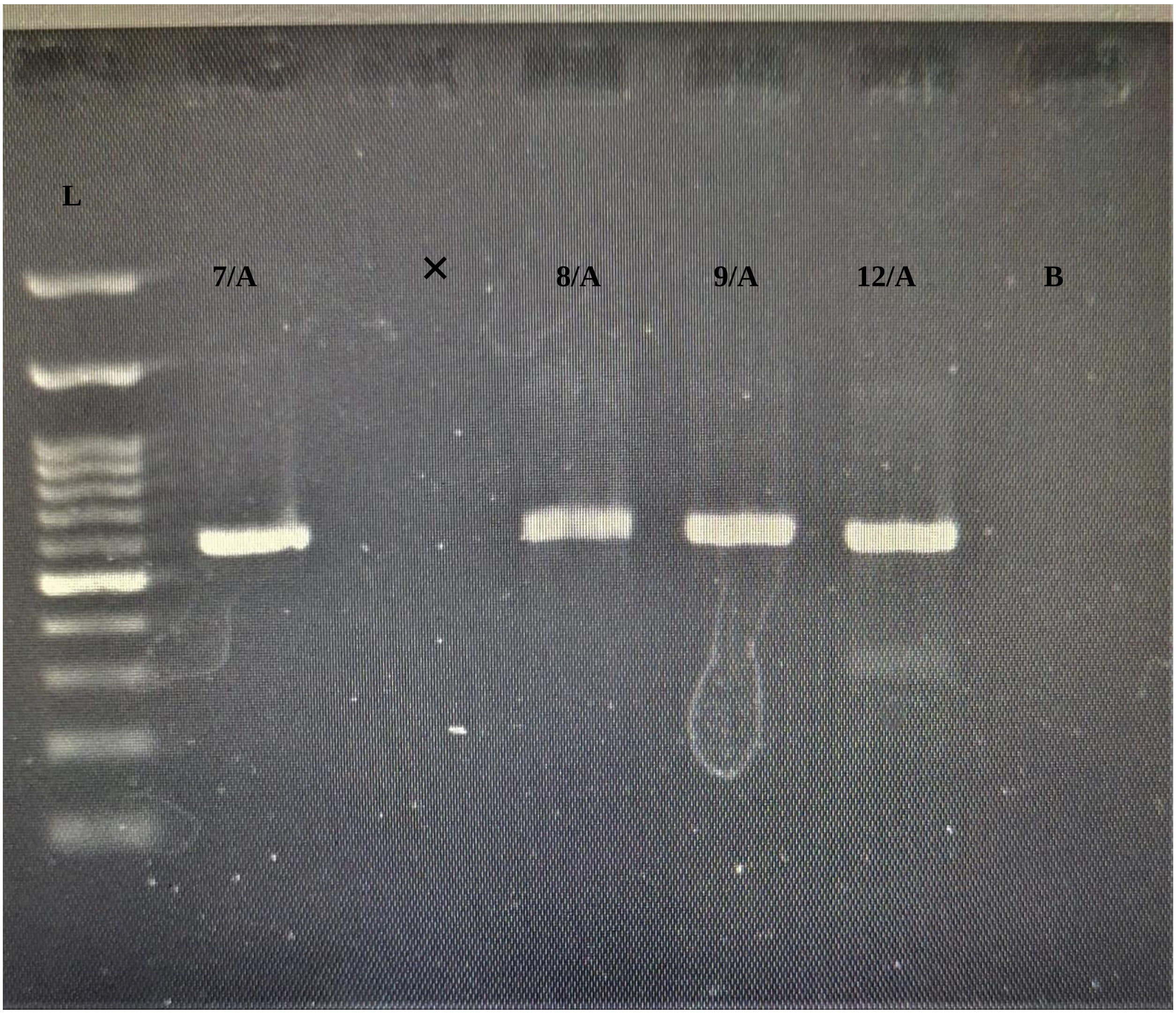
L
7/A
×
8/A
9/A
12/A
B

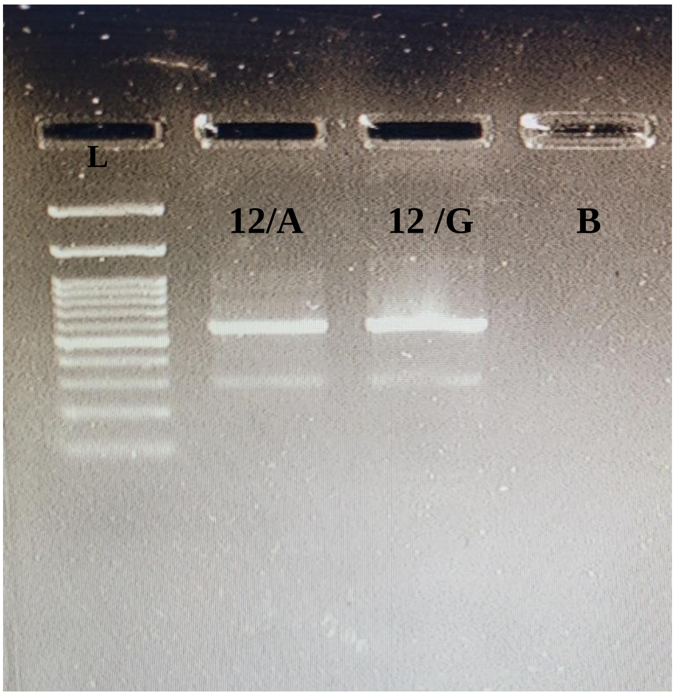
L
12/A
12 /G
B

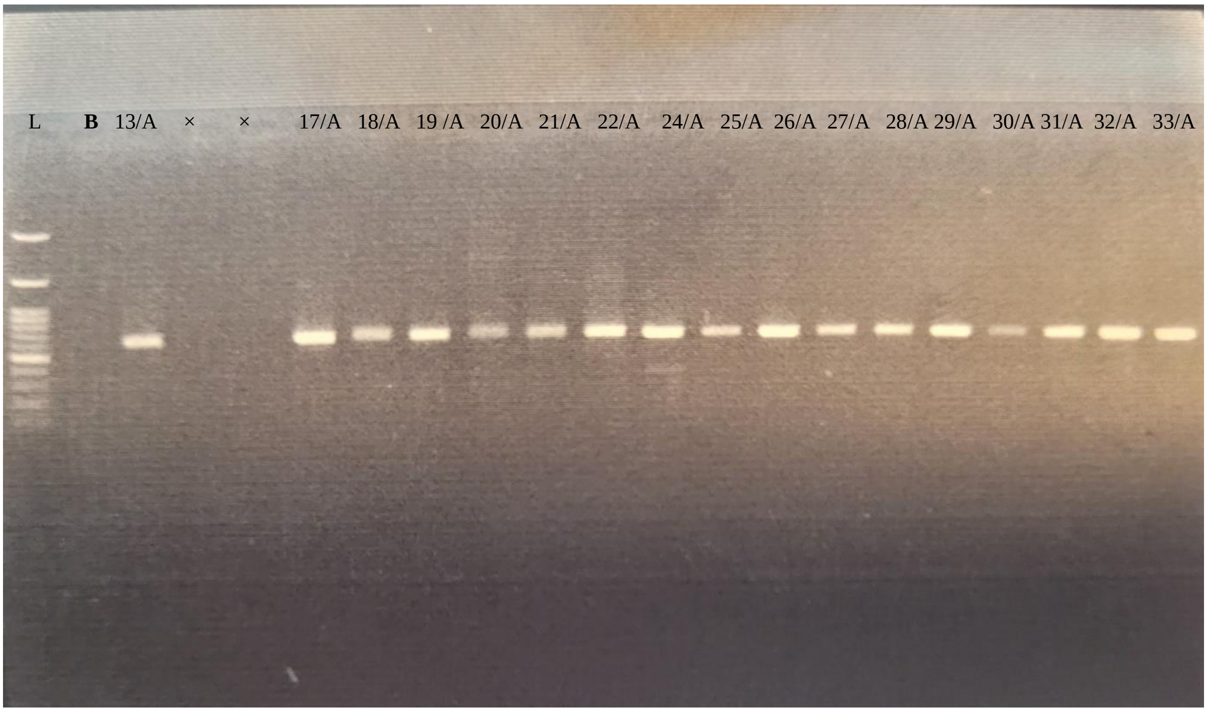
L
B
13/A
×
×
17/A
18/A
19 /A
20/A
21/A
22/A
24/A
25/A
26/A
27/A
28/A
29/A
30/A
31/A
32/A
33/A

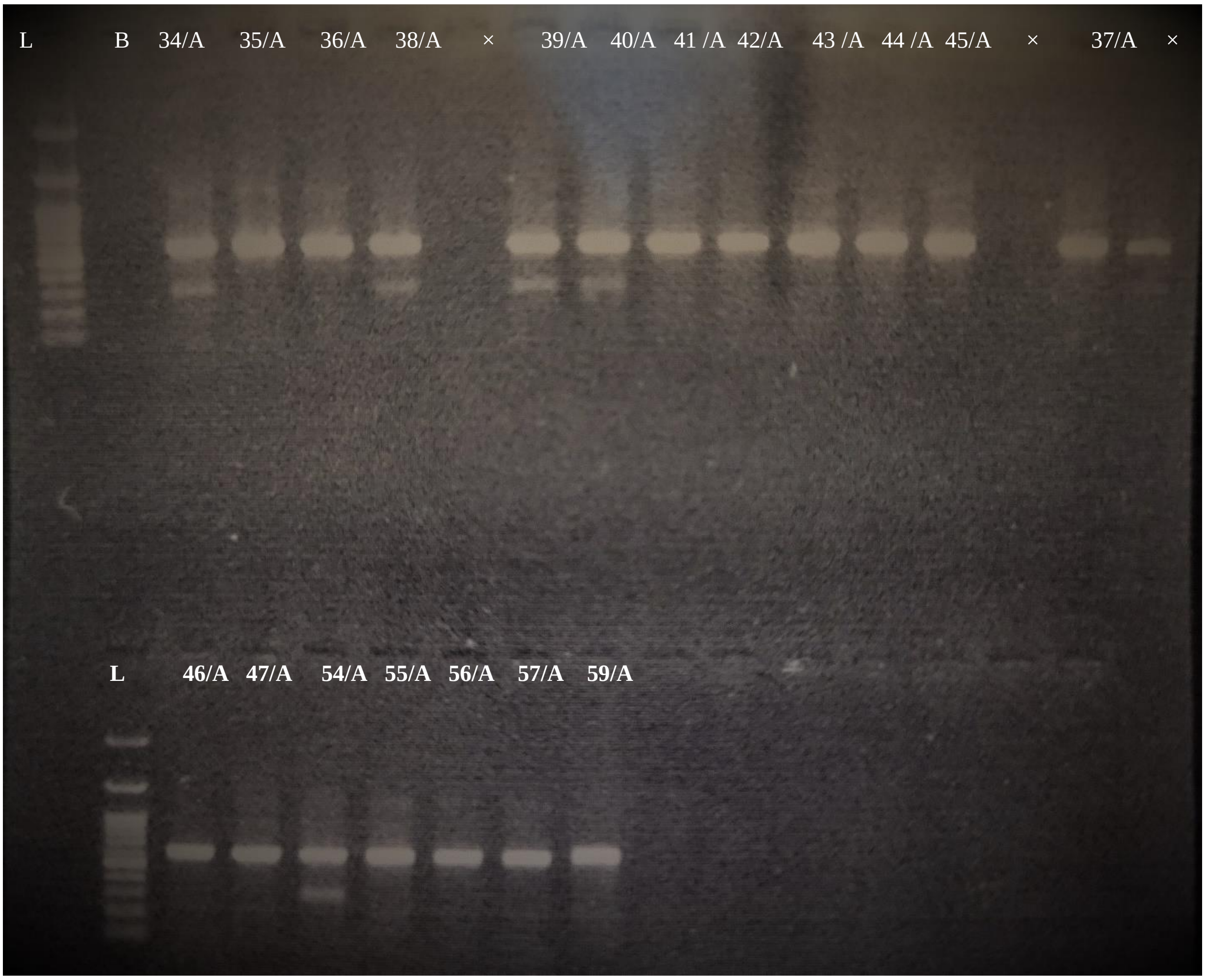
L
B
34/A
35/A
36/A
38/A
×
39/A
40/A
41 /A
42/A
43 /A
44 /A
45/A
×
37/A
×
L
46/A
47/A
54/A
55/A
56/A
57/A
59/A

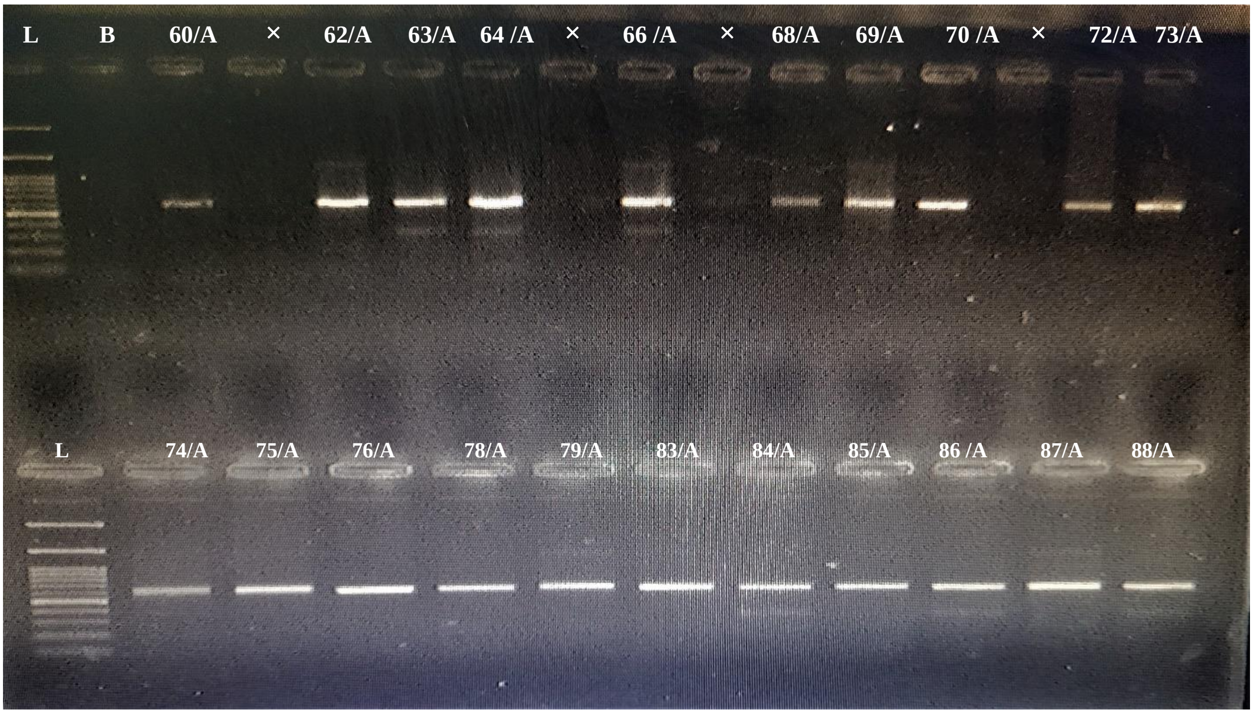
L B 60/A × 62/A 63/A 64 /A × 66 /A × 68/A 69/A 70 /A × 72/A 73/A
L 74/A 75/A 76/A 78/A 79/A 83/A 84/A 85/A 86 /A 87/A 88/A

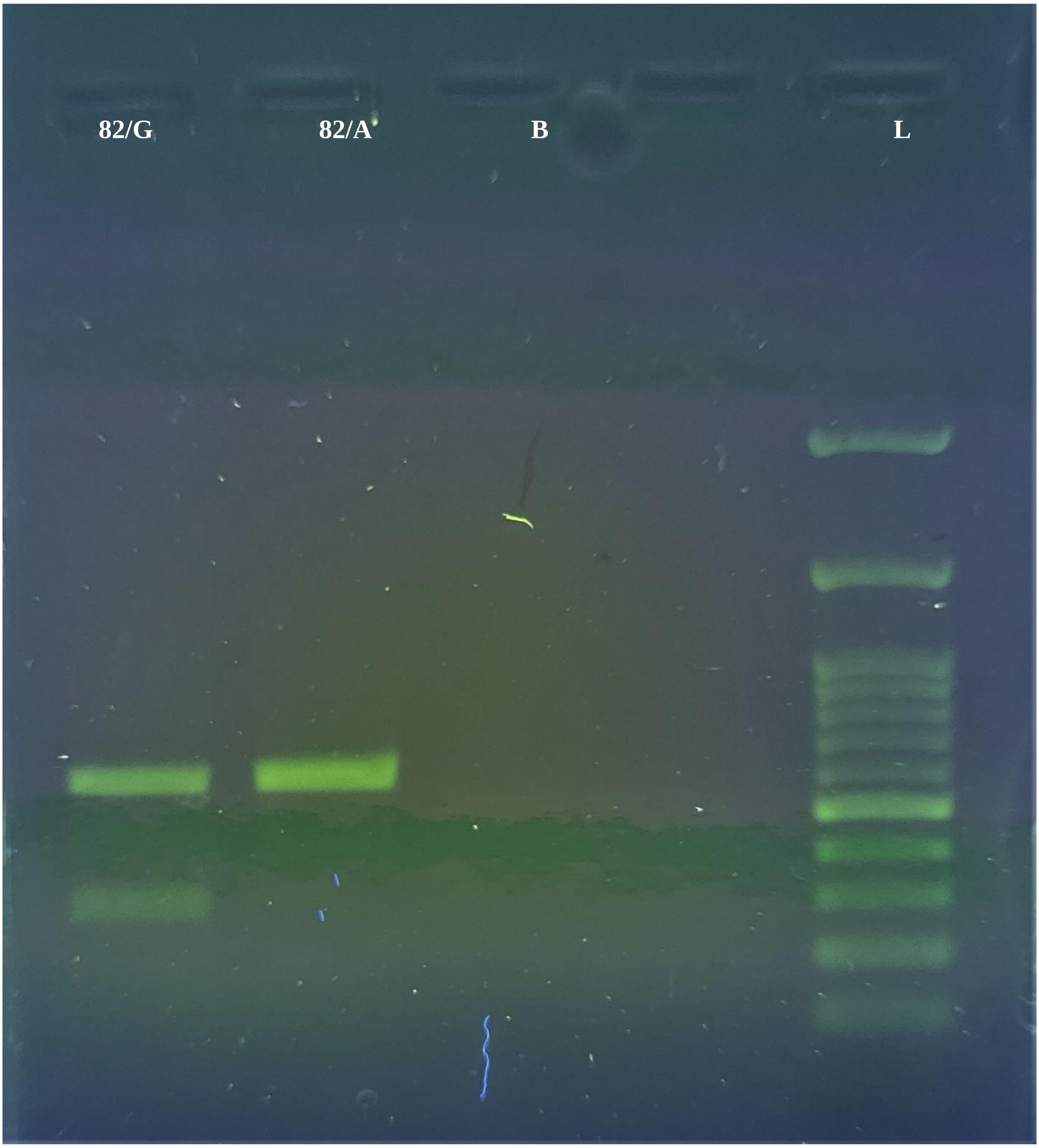
82/G
82/A
B
L

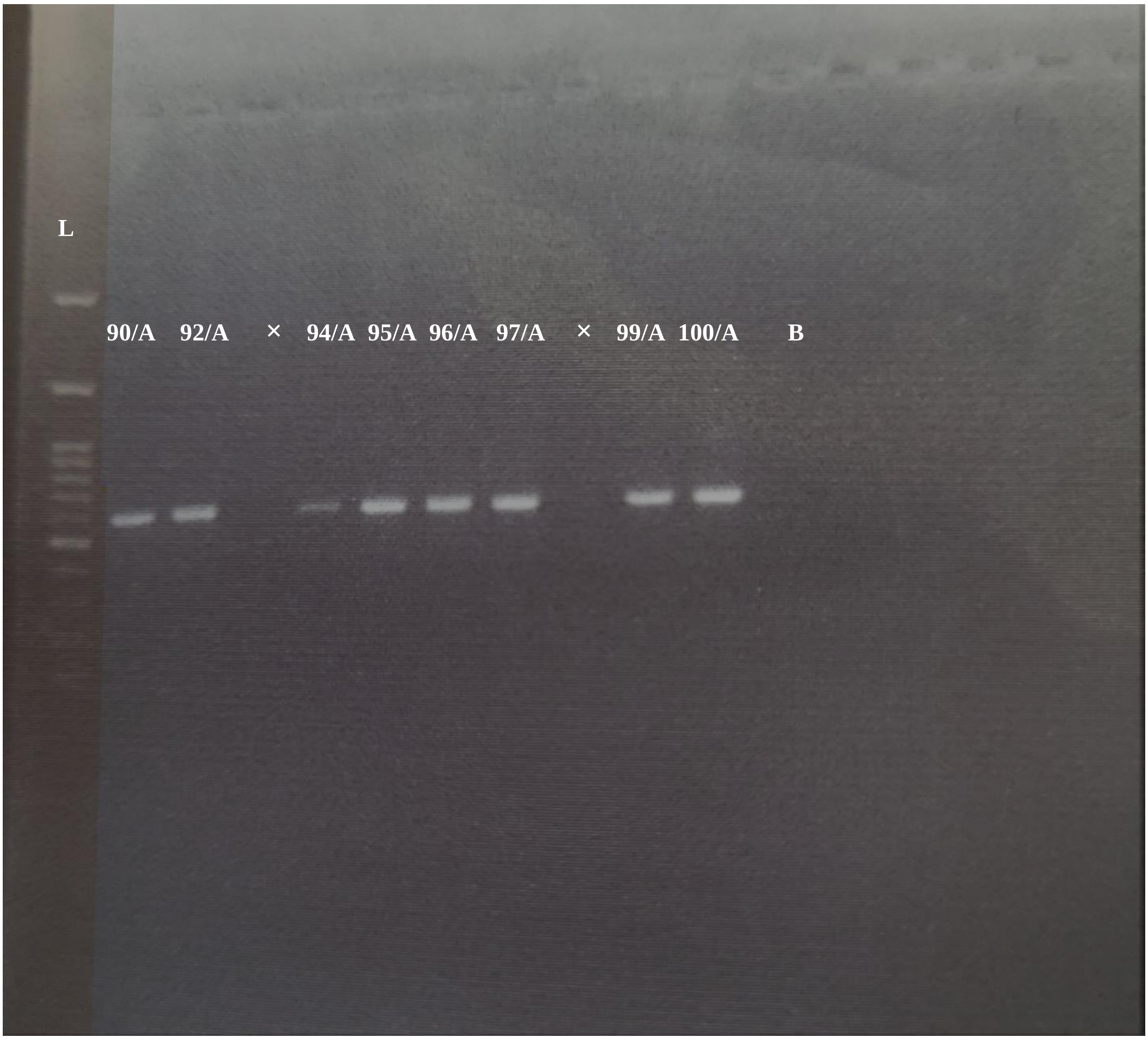
L
90/A
92/A
×
94/A
95/A
96/A
97/A
×
99/A
100/A
B

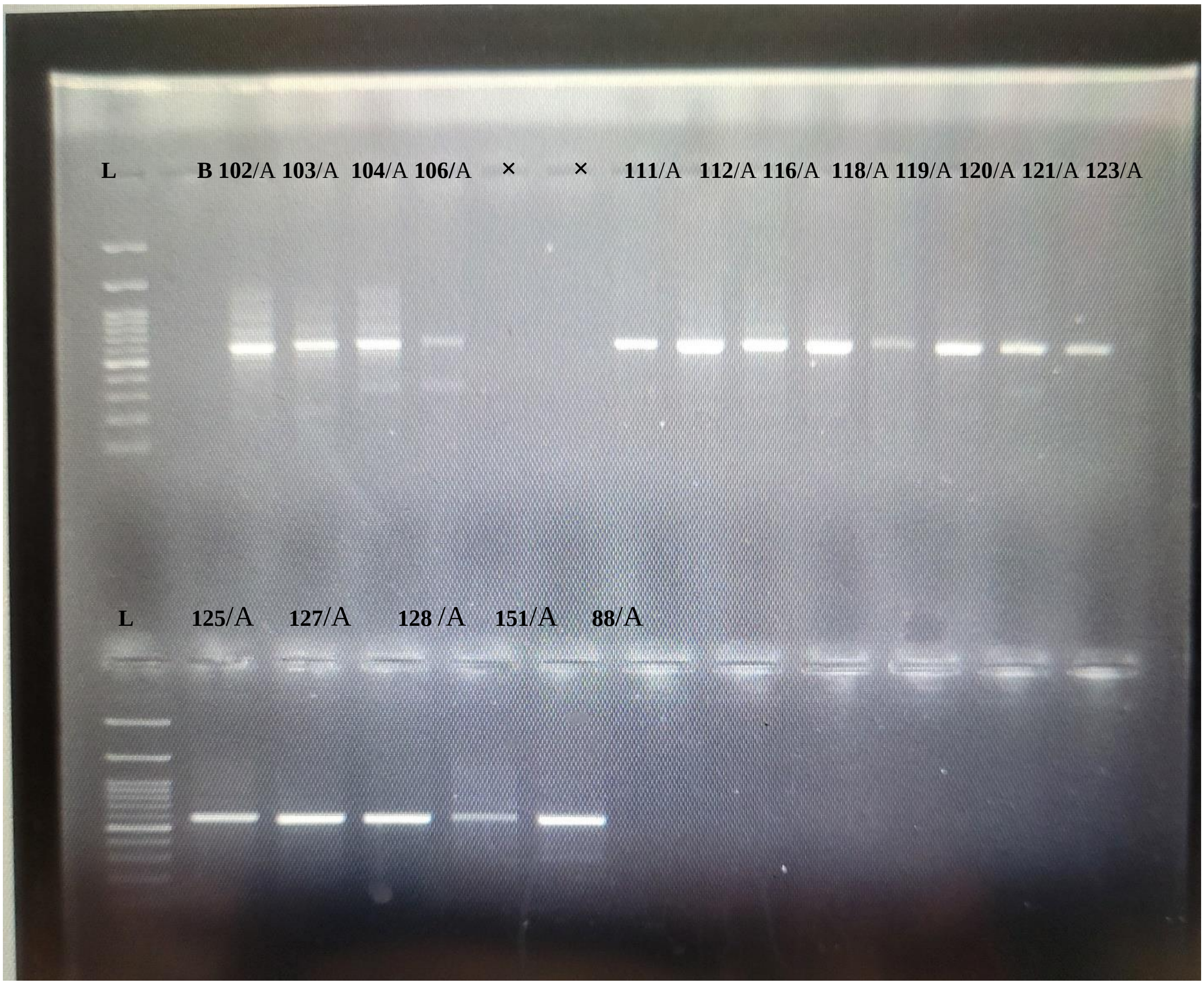
L B 102/A 103/A 104/A 106/A × × 111/A 112/A 116/A 118/A 119/A 120/A 121/A 123/A
L 125/A 127/A 128 /A 151/A 88/A

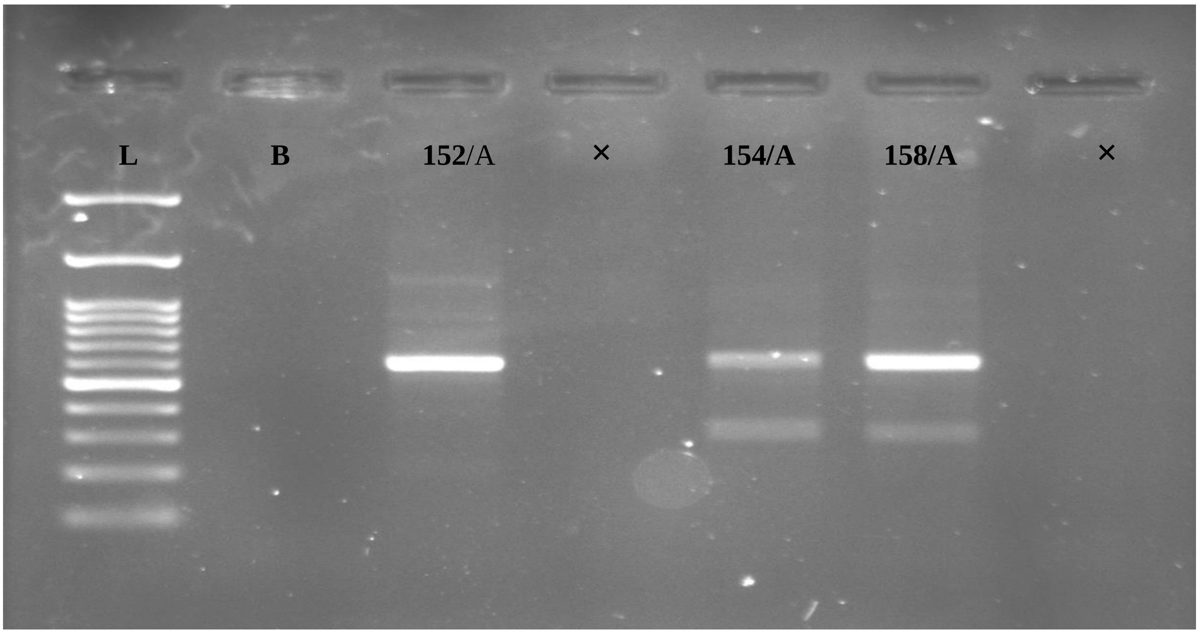
L
B
152/A
×
154/A
158/A
×

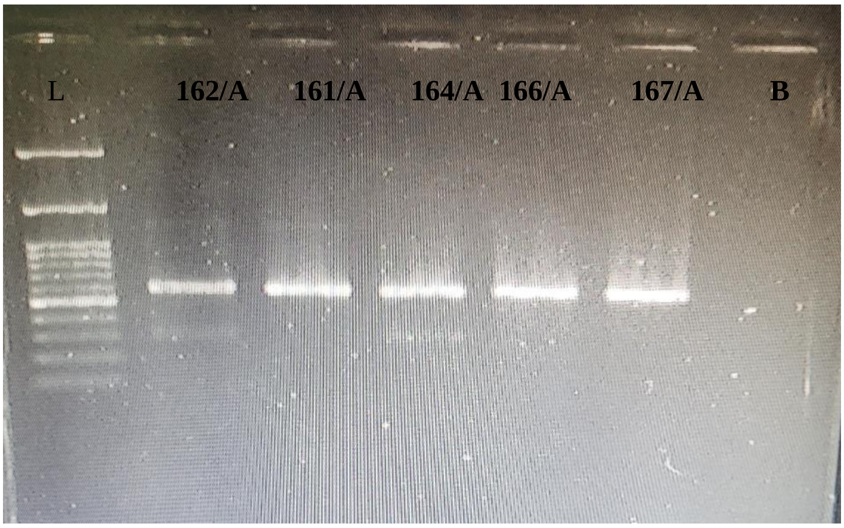
L
162/A
161/A
164/A
166/A
167/A
B

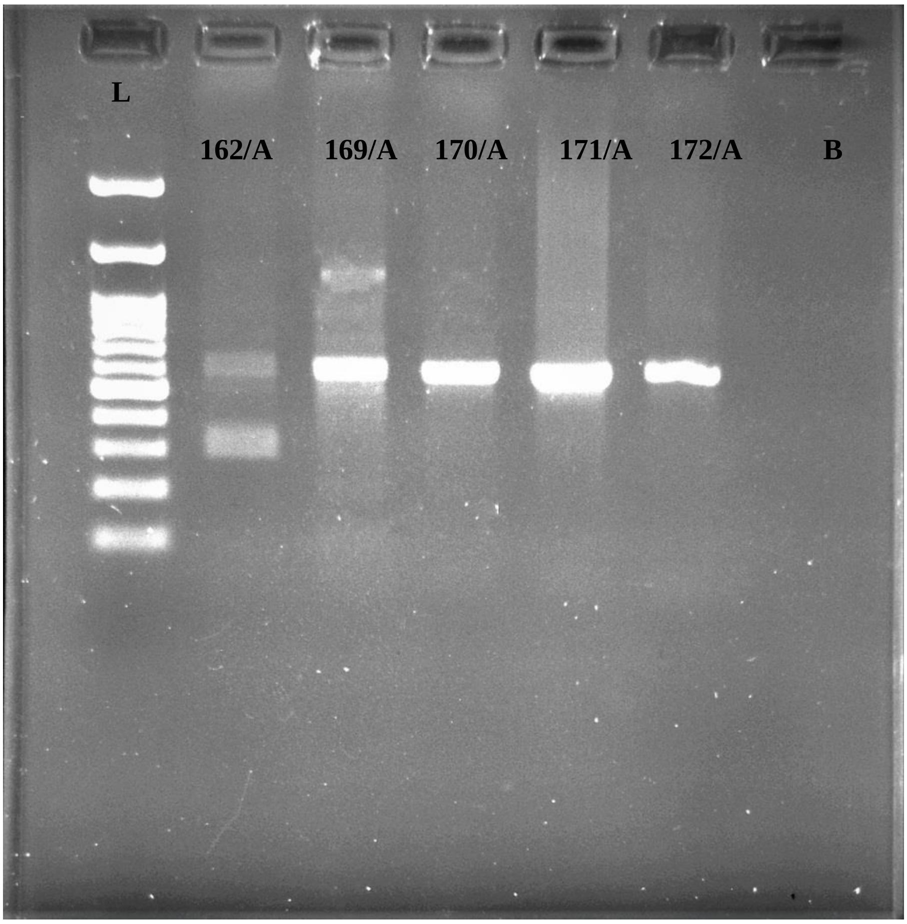
L
162/A
169/A
170/A
171/A
172/A
B

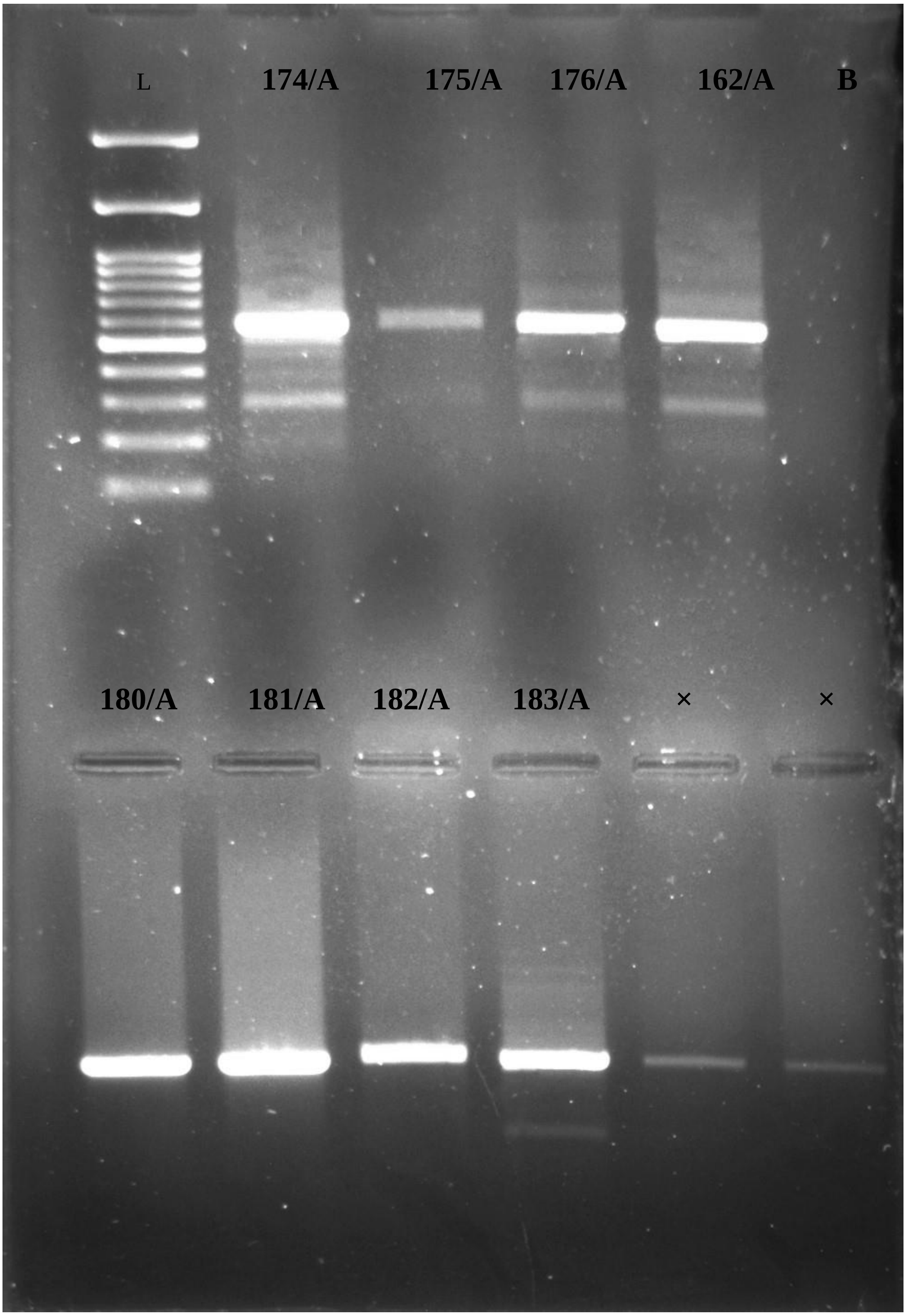
L
174/A
175/A
176/A
162/A
B
180/A
181/A
182/A
183/A
×
×

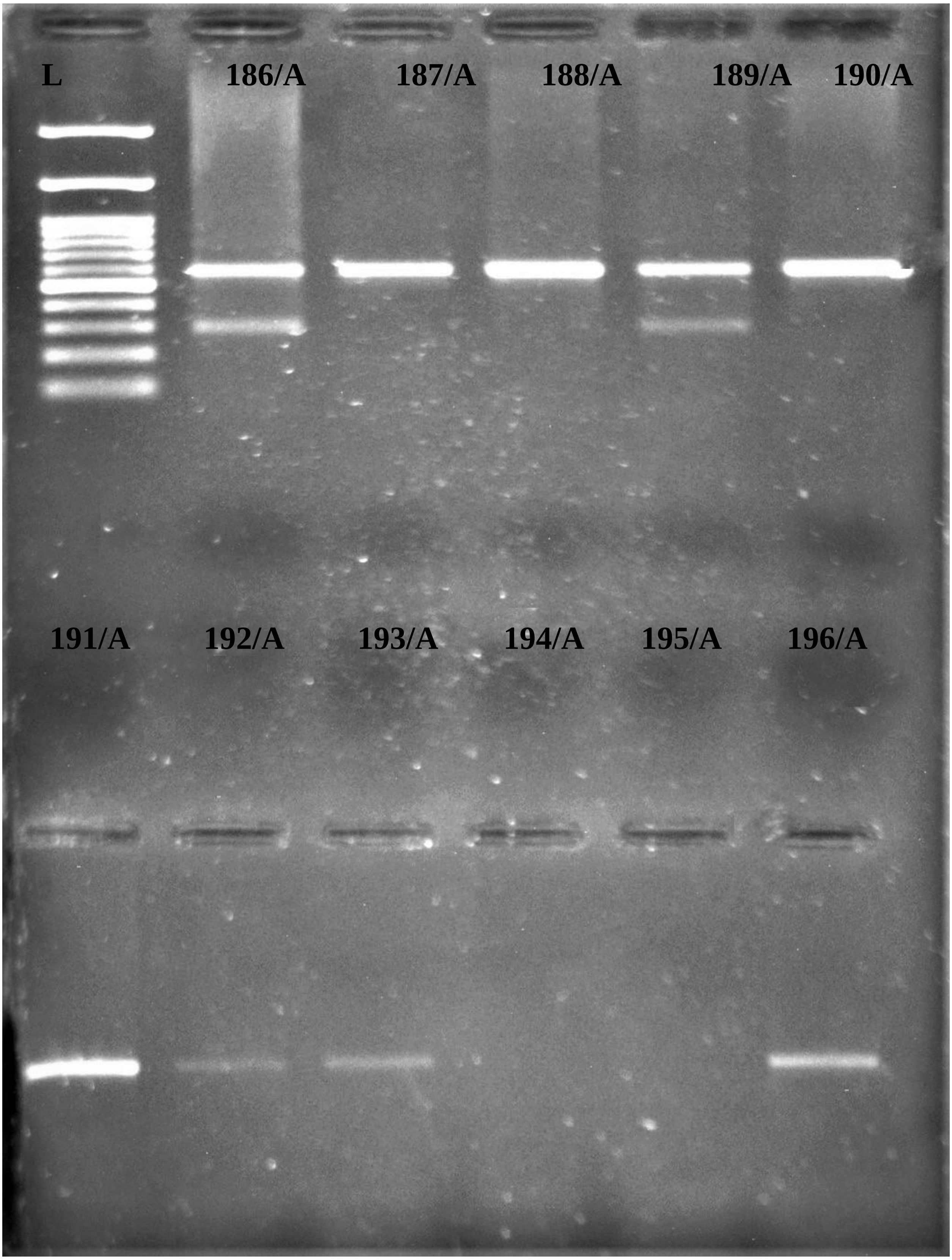
L
186/A
187/A
188/A
189/A
190/A
191/A
192/A
193/A
194/A
195/A
196/A

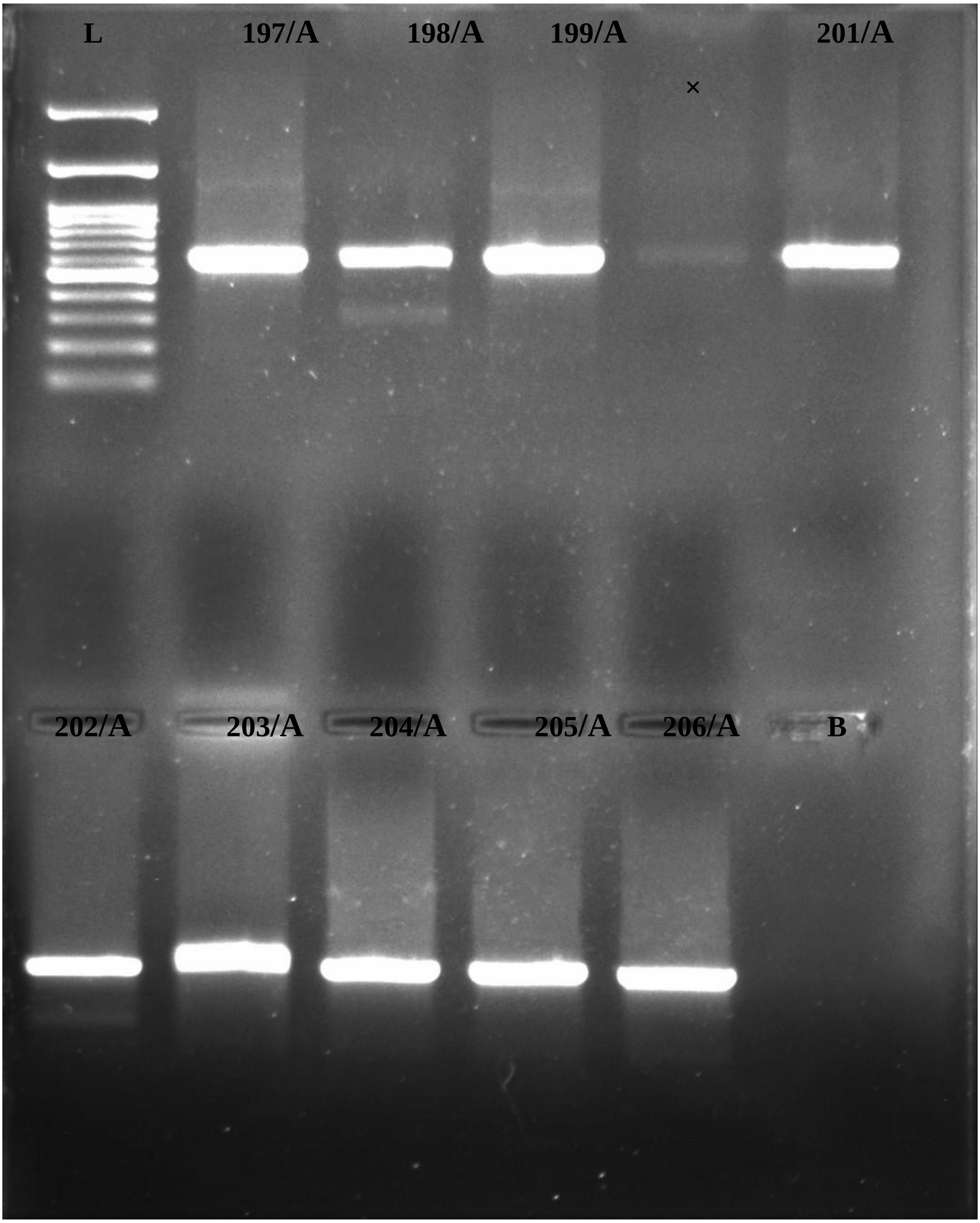

L
197/A
198/A
199/A
×
201/A
202/A
203/A
204/A
205/A
206/A
B

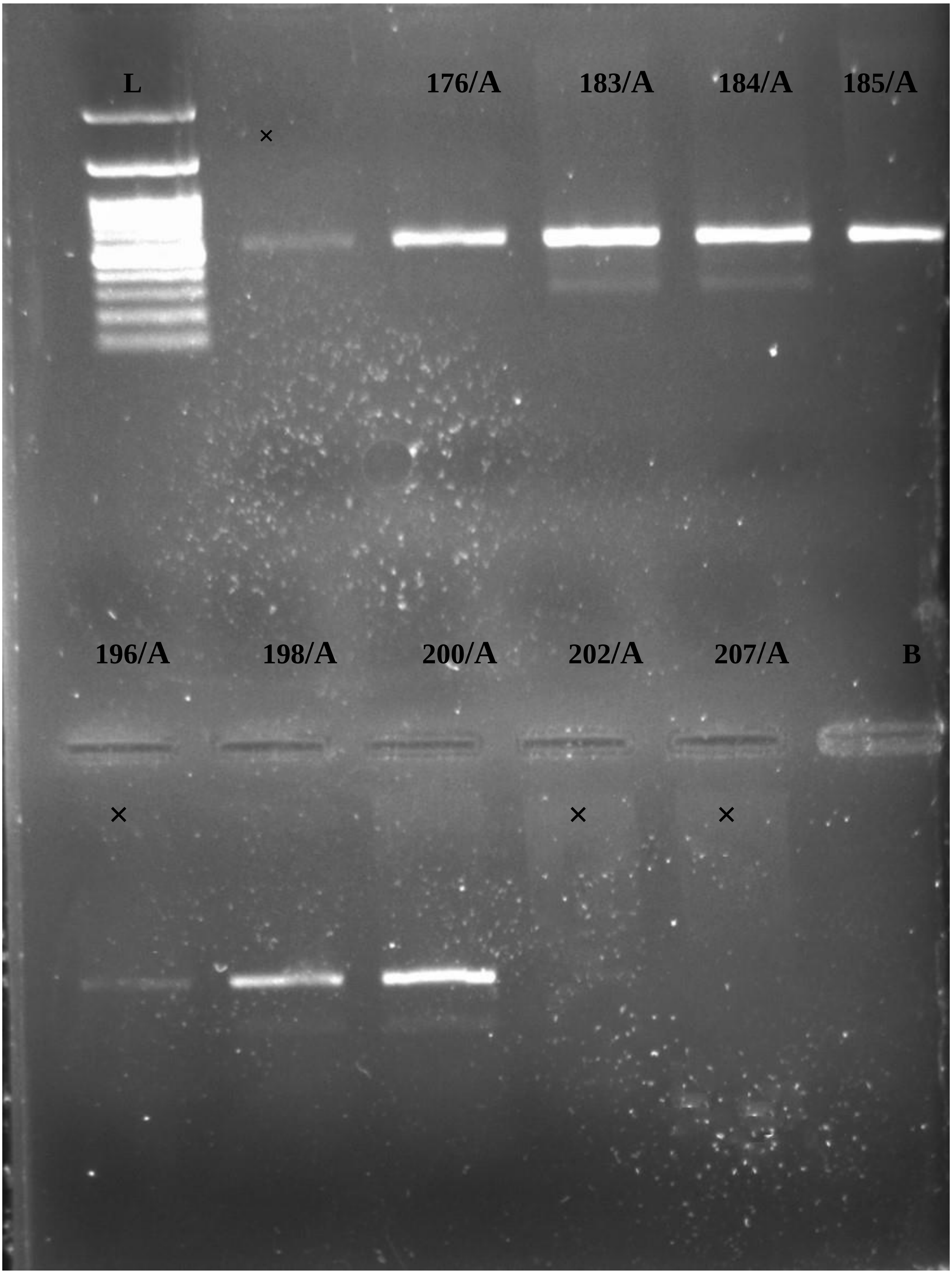
L
×
176/A
183/A
184/A
185/A
196/A
198/A
200/A
202/A
207/A
B
×
×
×

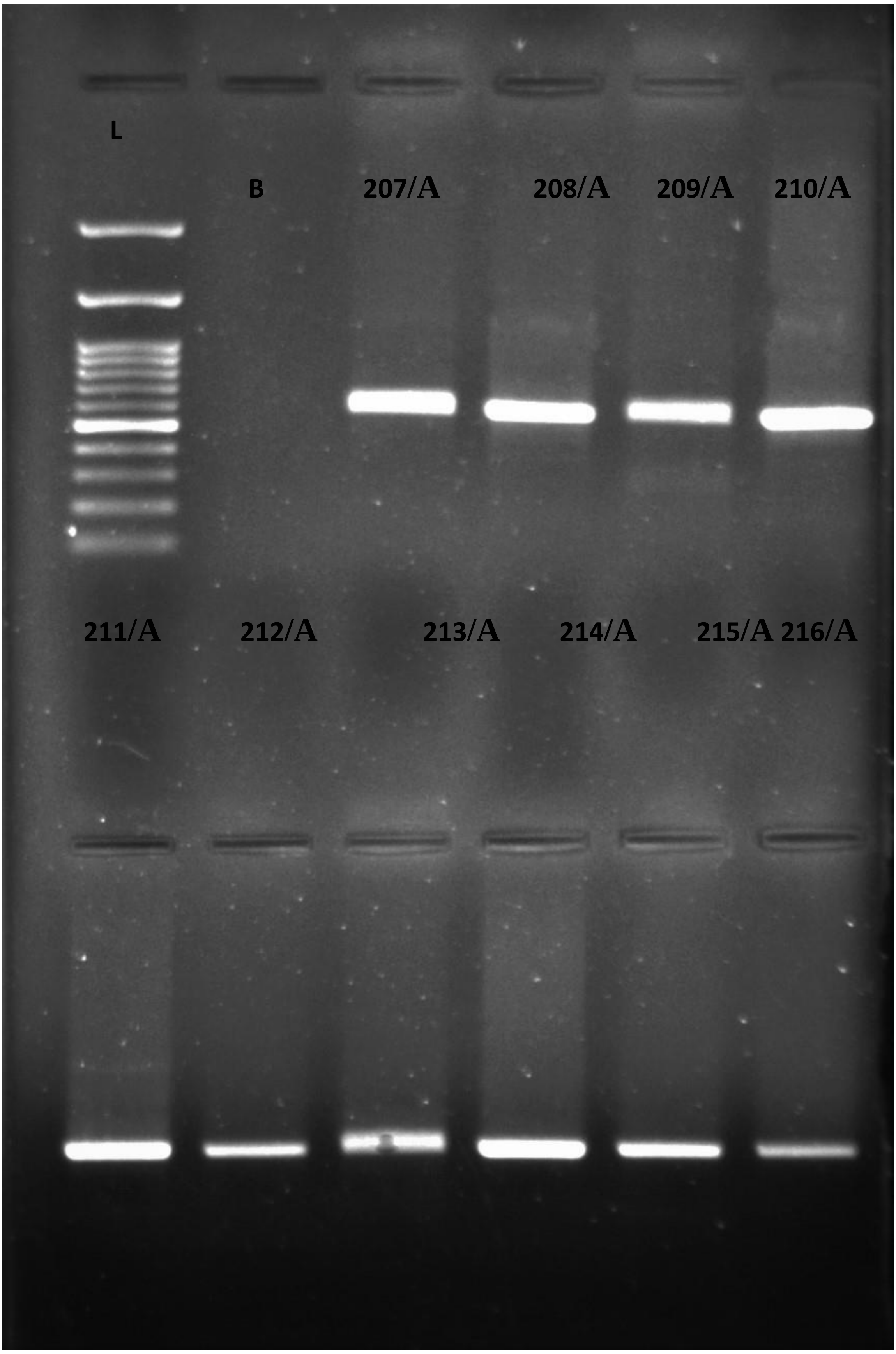
L
B
207/A
208/A
209/A
210/A
211/A
212/A
213/A
214/A
215/A
216/A

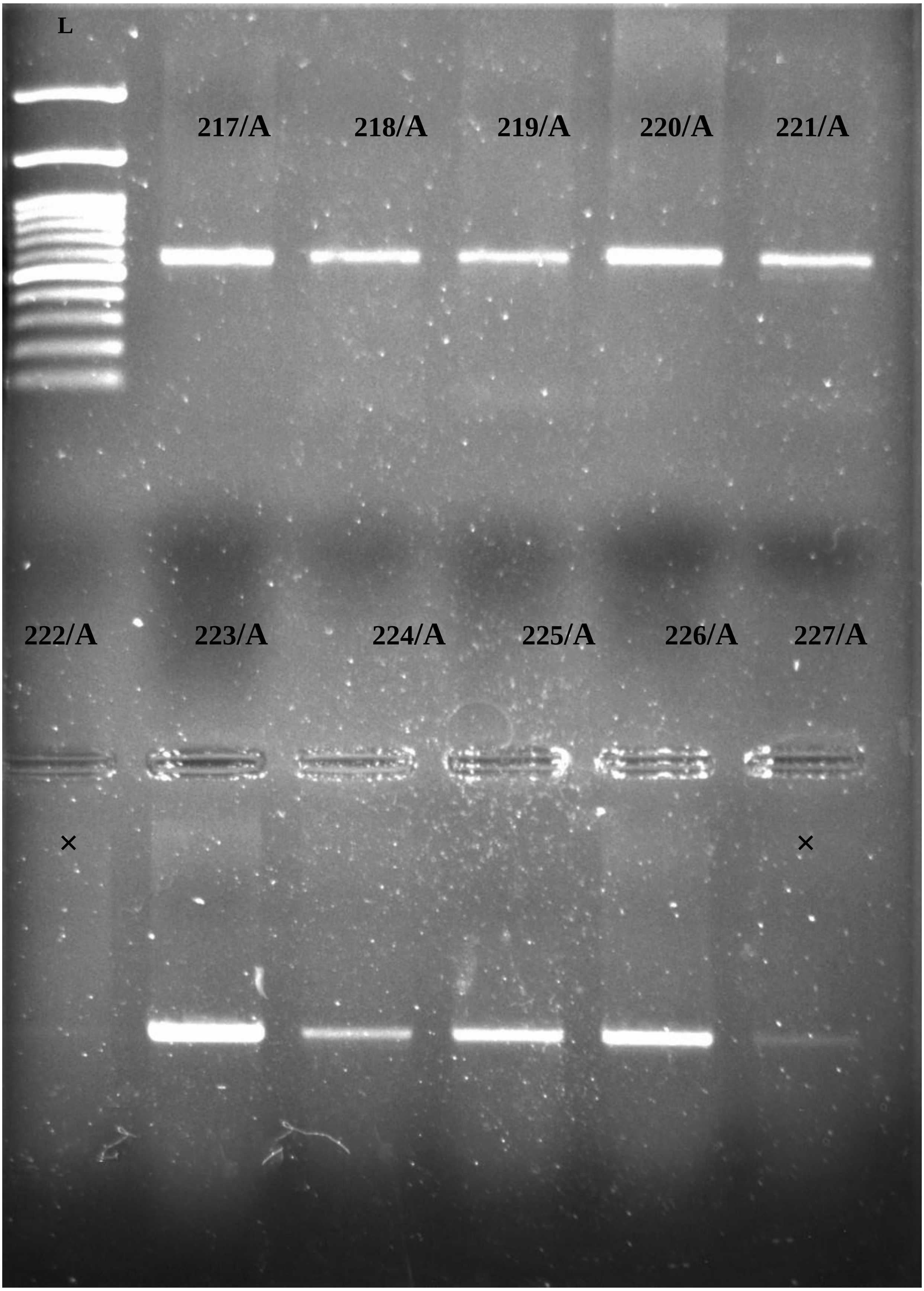
L
217/A
218/A
219/A
220/A
221/A
222/A
223/A
224/A
225/A
226/A
227/A
×
×

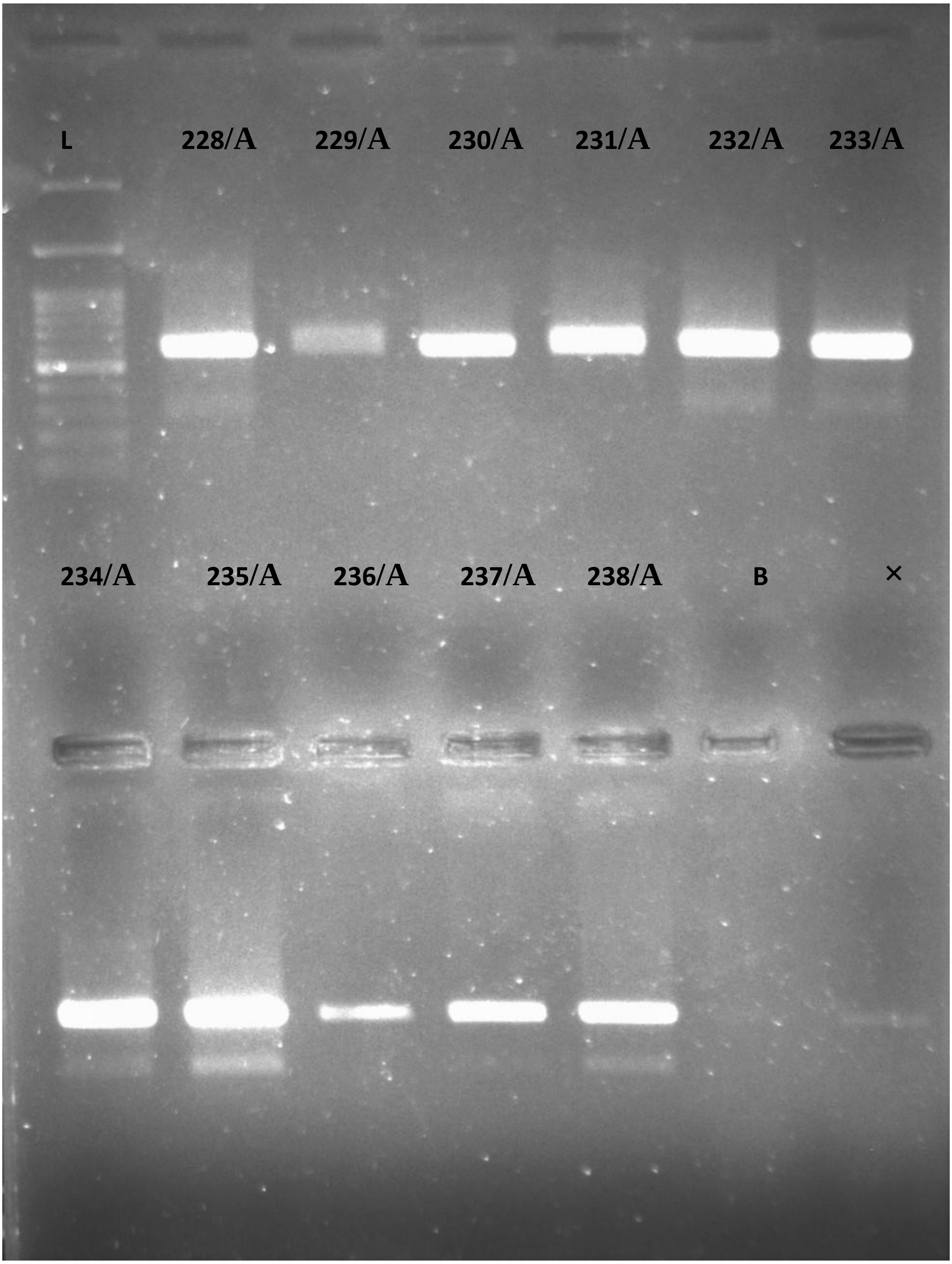
L
228/A
229/A
230/A
231/A
232/A
233/A
234/A
235/A
236/A
237/A
238/A
B
×

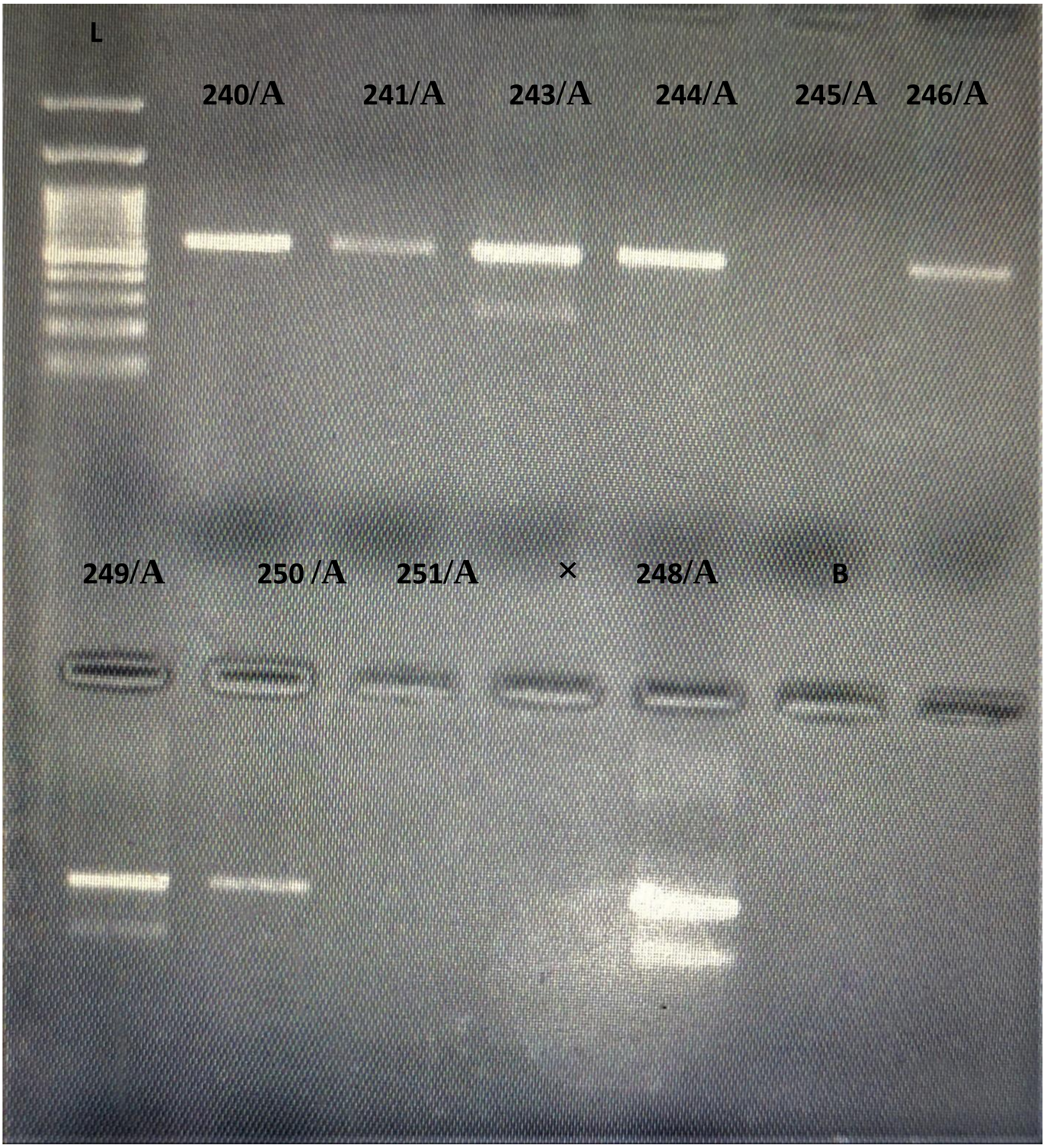
L
240/A
241/A
243/A
244/A
245/A
246/A
249/A
250 /A
251/A
×
248/A
B

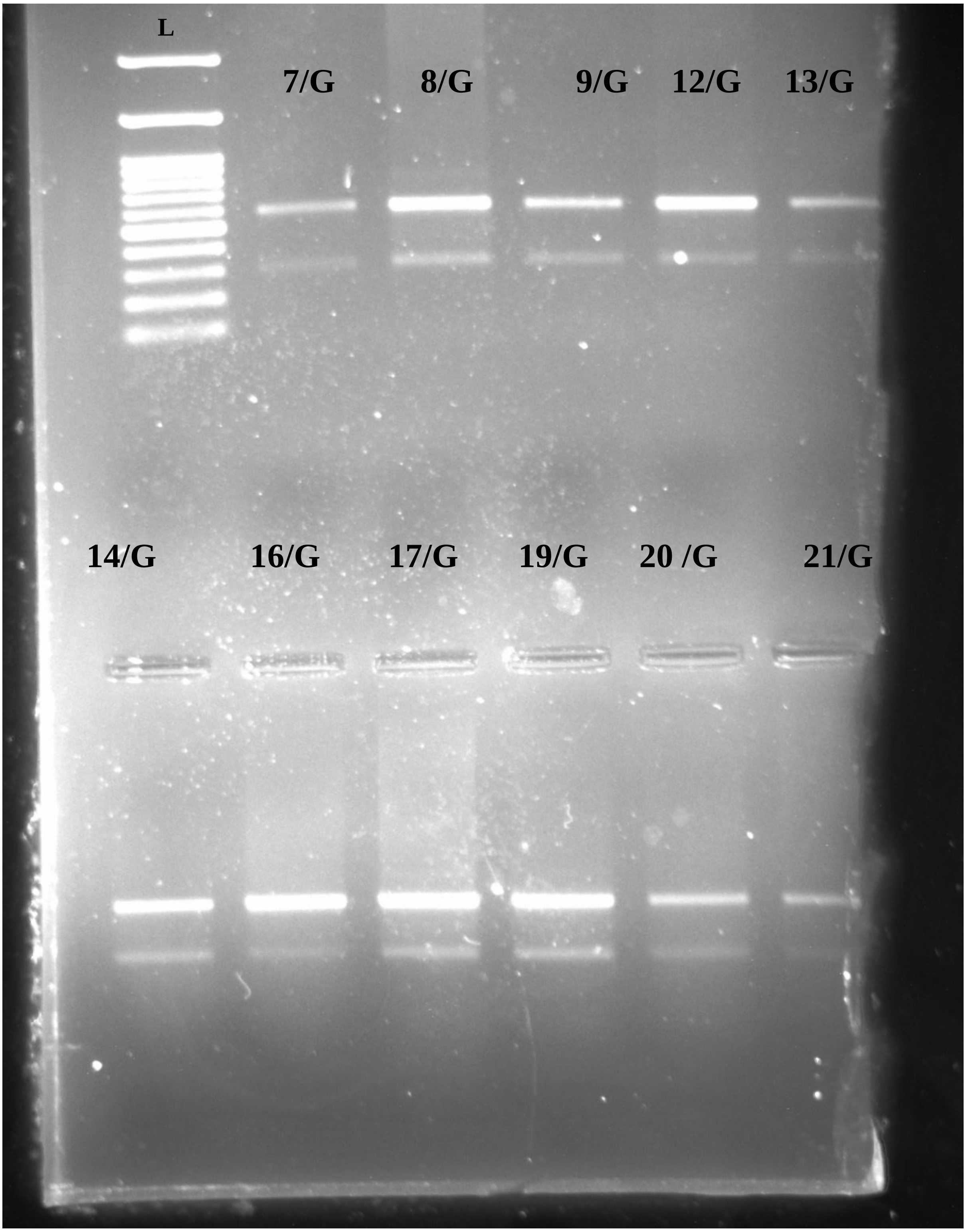
L
7/G
8/G
9/G
12/G
13/G
14/G
16/G
17/G
19/G
20 /G
21/G

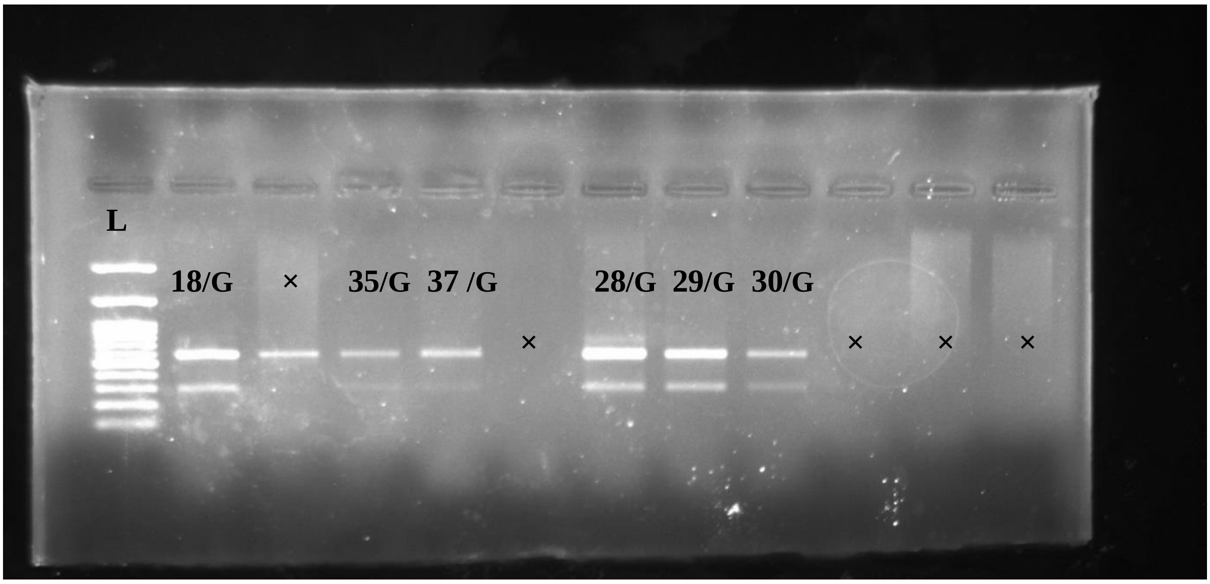
L
18/G
×
35/G
37 /G
×
28/G
29/G
30/G
×
×
×

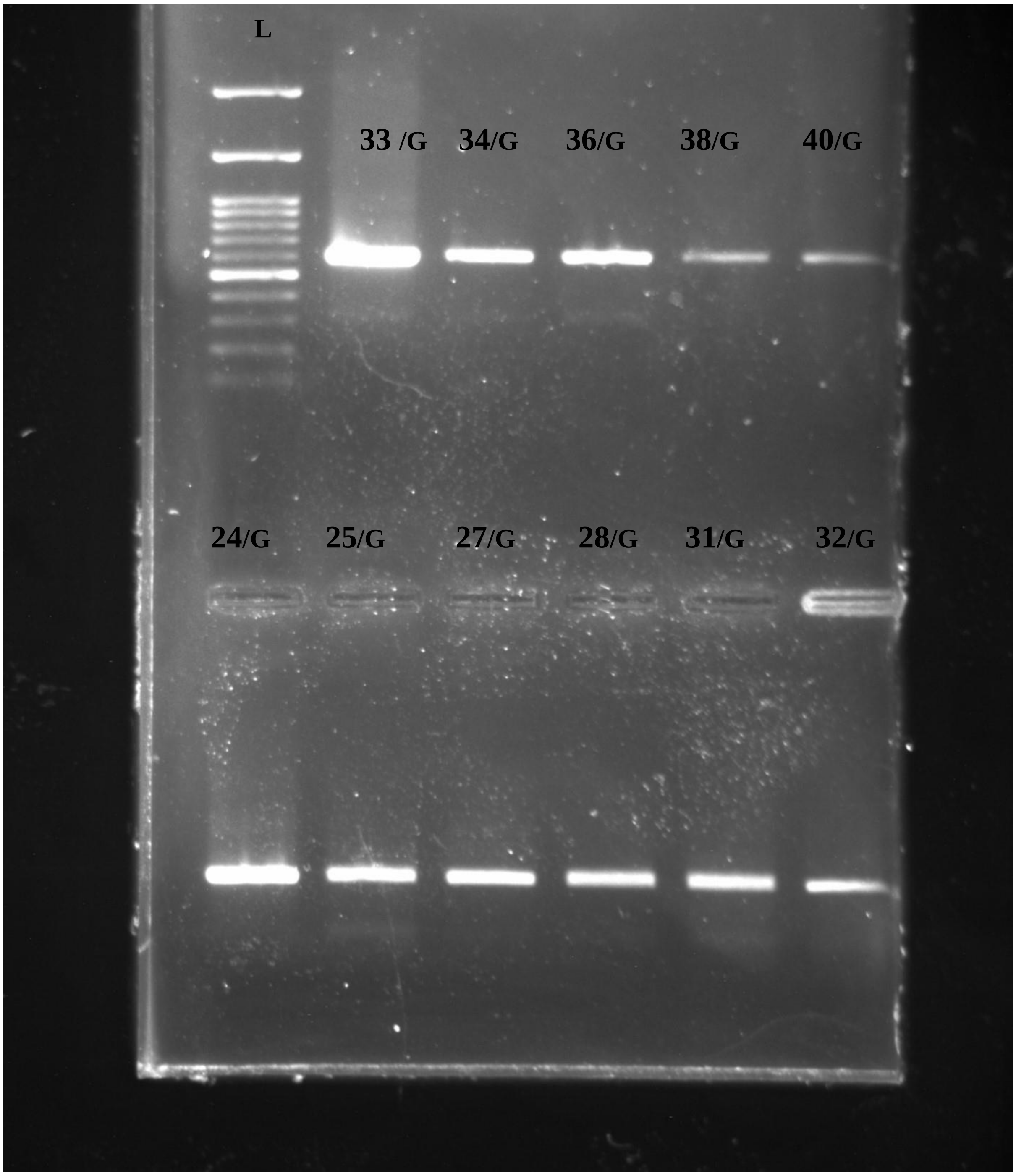
L
33 /G
34/G
36/G
38/G
40/G
24/G
25/G
27/G
28/G
31/G
32/G

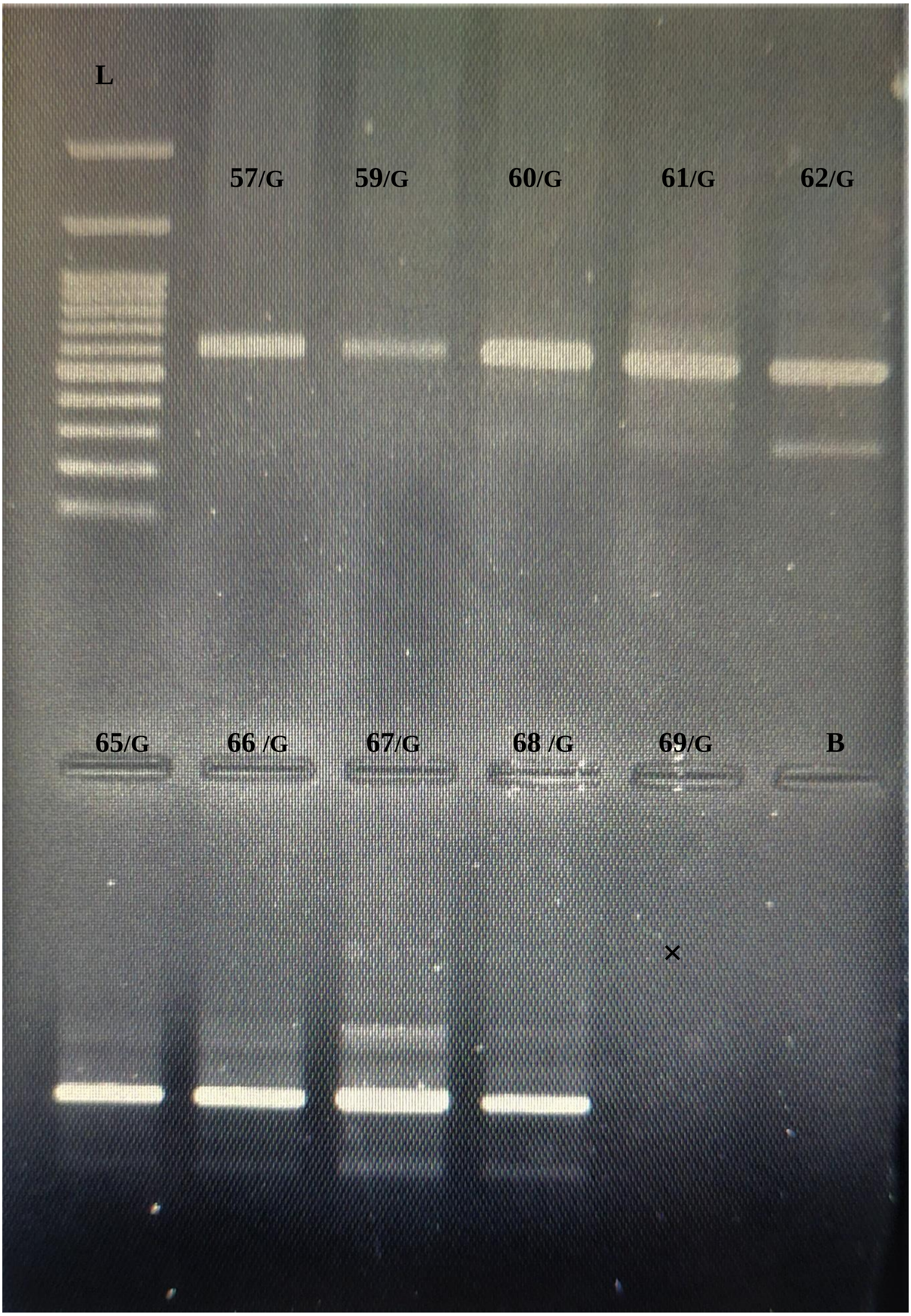
L
57/G
59/G
60/G
61/G
62/G
65/G
66 /G
67/G
68 /G
69/G
B
×

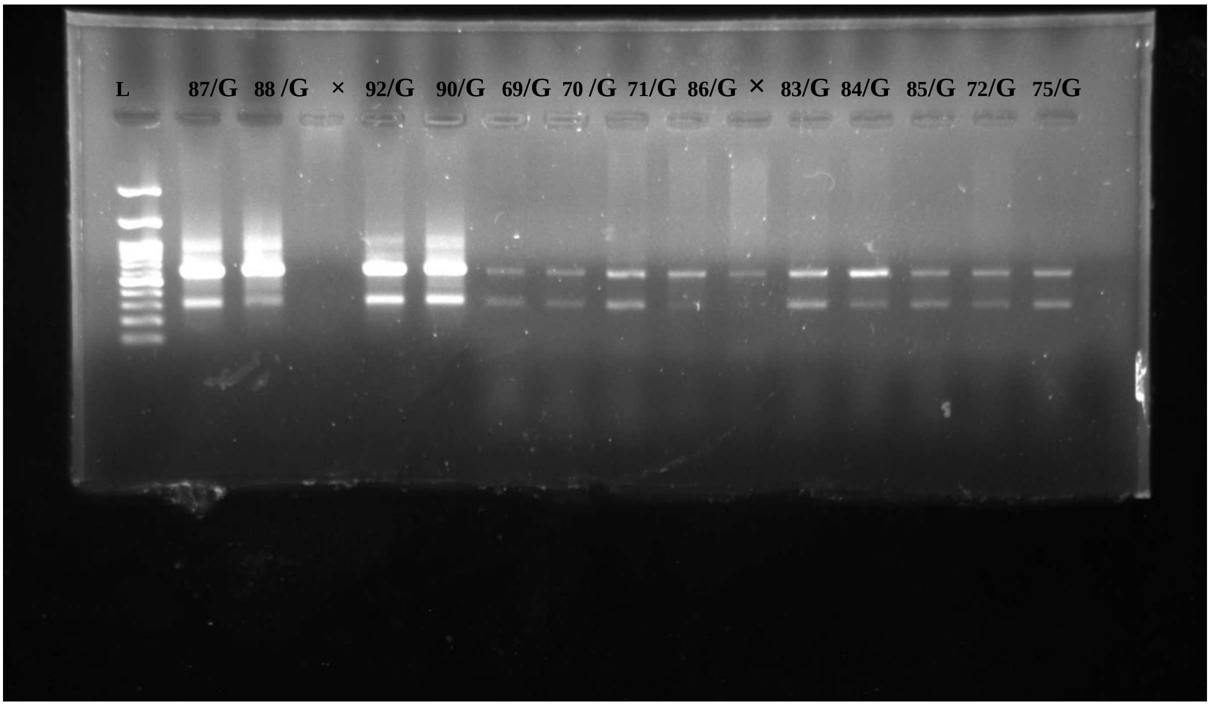
L
87/G
88 /G
×
92/G
90/G
69/G
70 /G
71/G
86/G
×
83/G
84/G
85/G
72/G
75/G

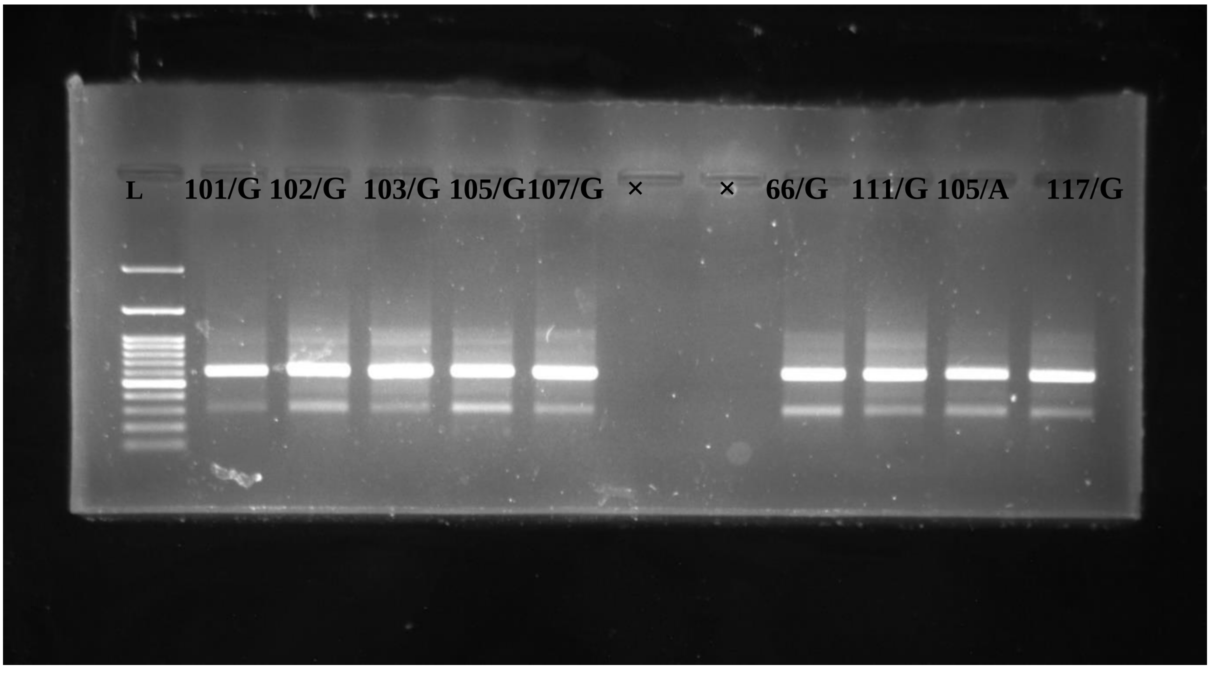
L
101/G
102/G
103/G
105/G
107/G
×
×
66/G
111/G
105/A
117/G

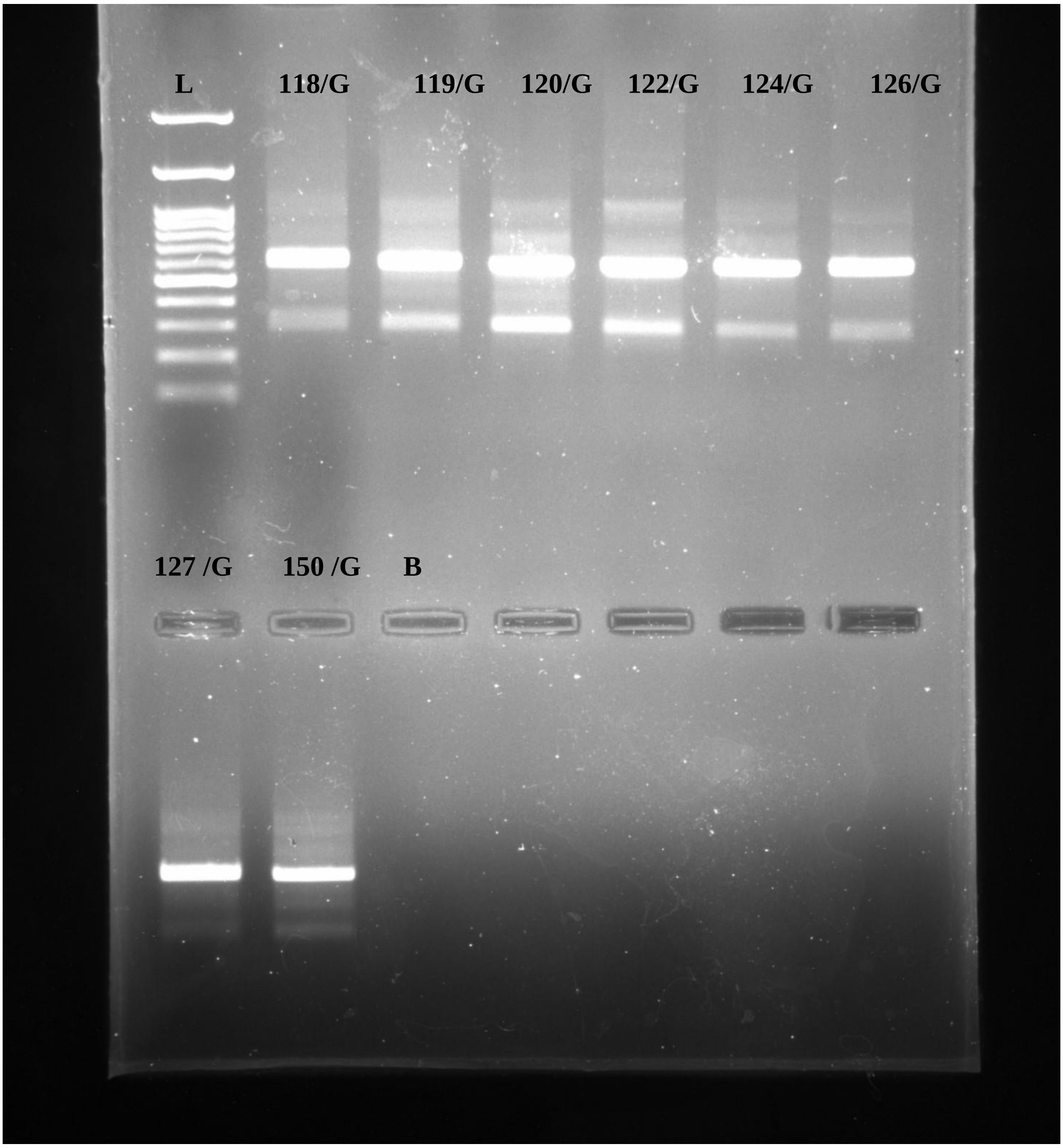
L
118/G
119/G
120/G
122/G
124/G
126/G
127 /G
150 /G
B

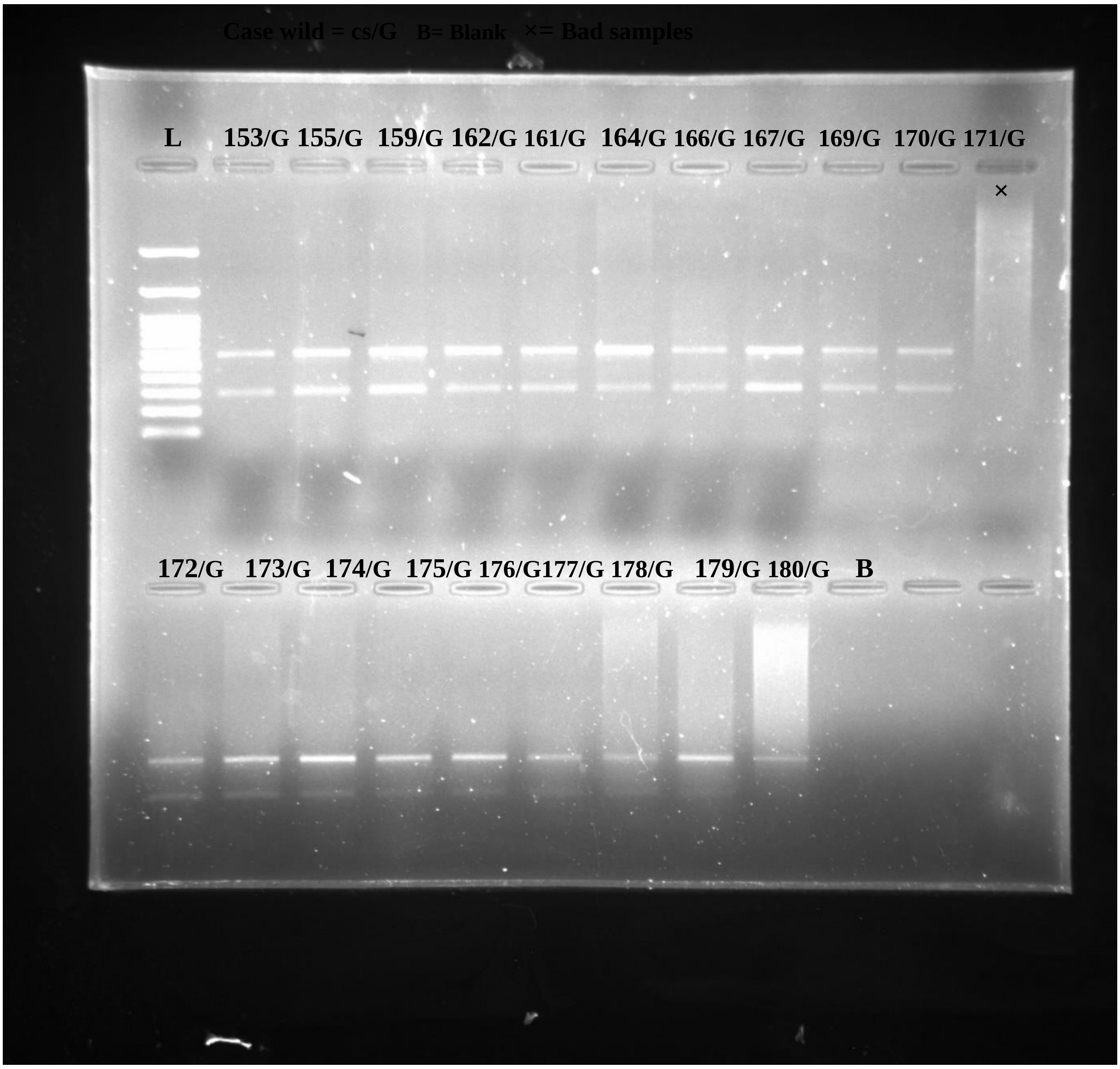
Case wild = cs/G B= Blank ×= Bad samples
L 153/G 155/G 159/G 162/G 161/G 164/G 166/G 167/G 169/G 170/G 171/G
×
172/G 173/G 174/G 175/G 176/G177/G 178/G 179/G 180/G B

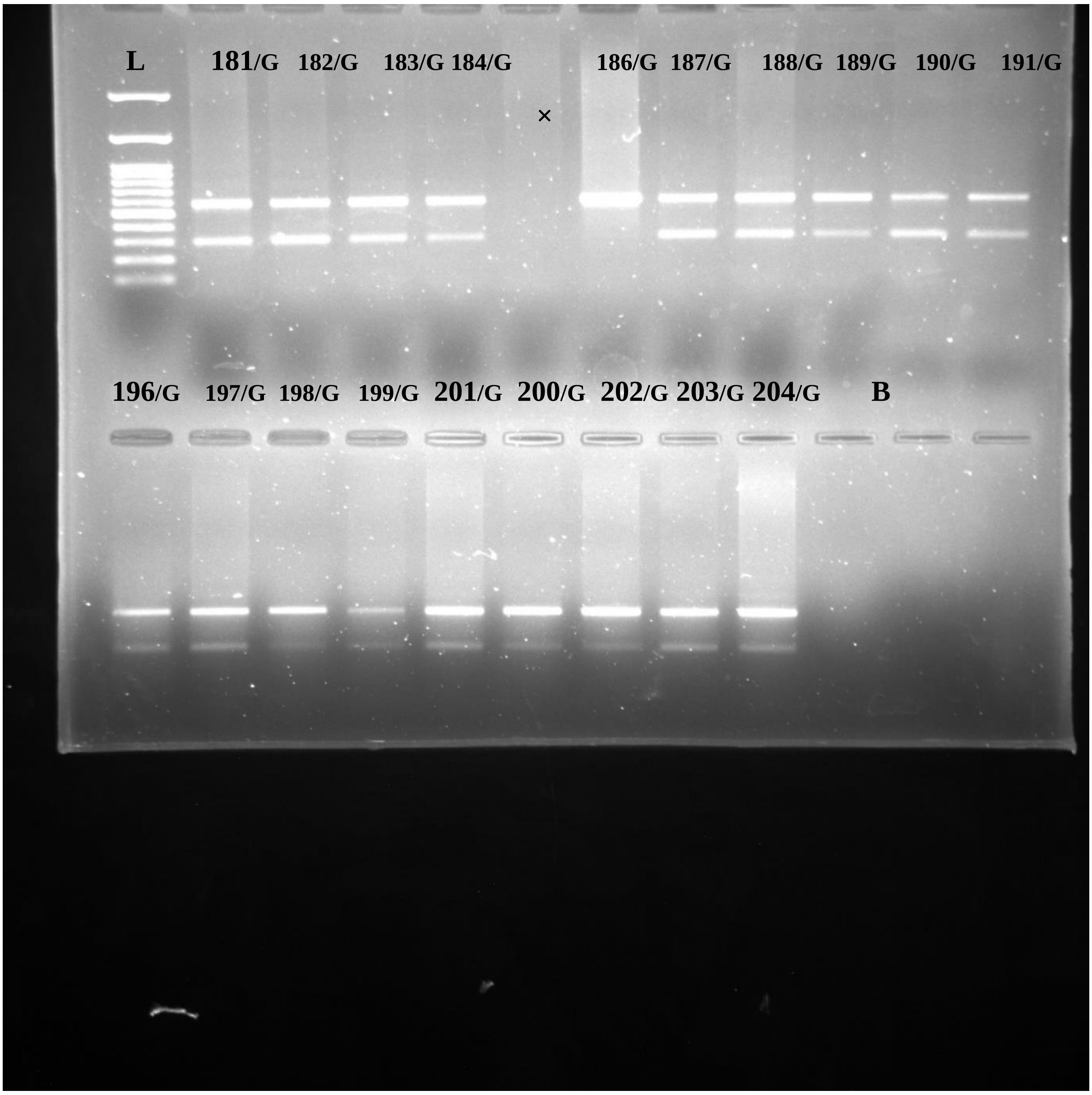
L
181/G
182/G
183/G
184/G
×
186/G
187/G
188/G
189/G
190/G
191/G
196/G
197/G
198/G
199/G
201/G
200/G
202/G
203/G
204/G
B

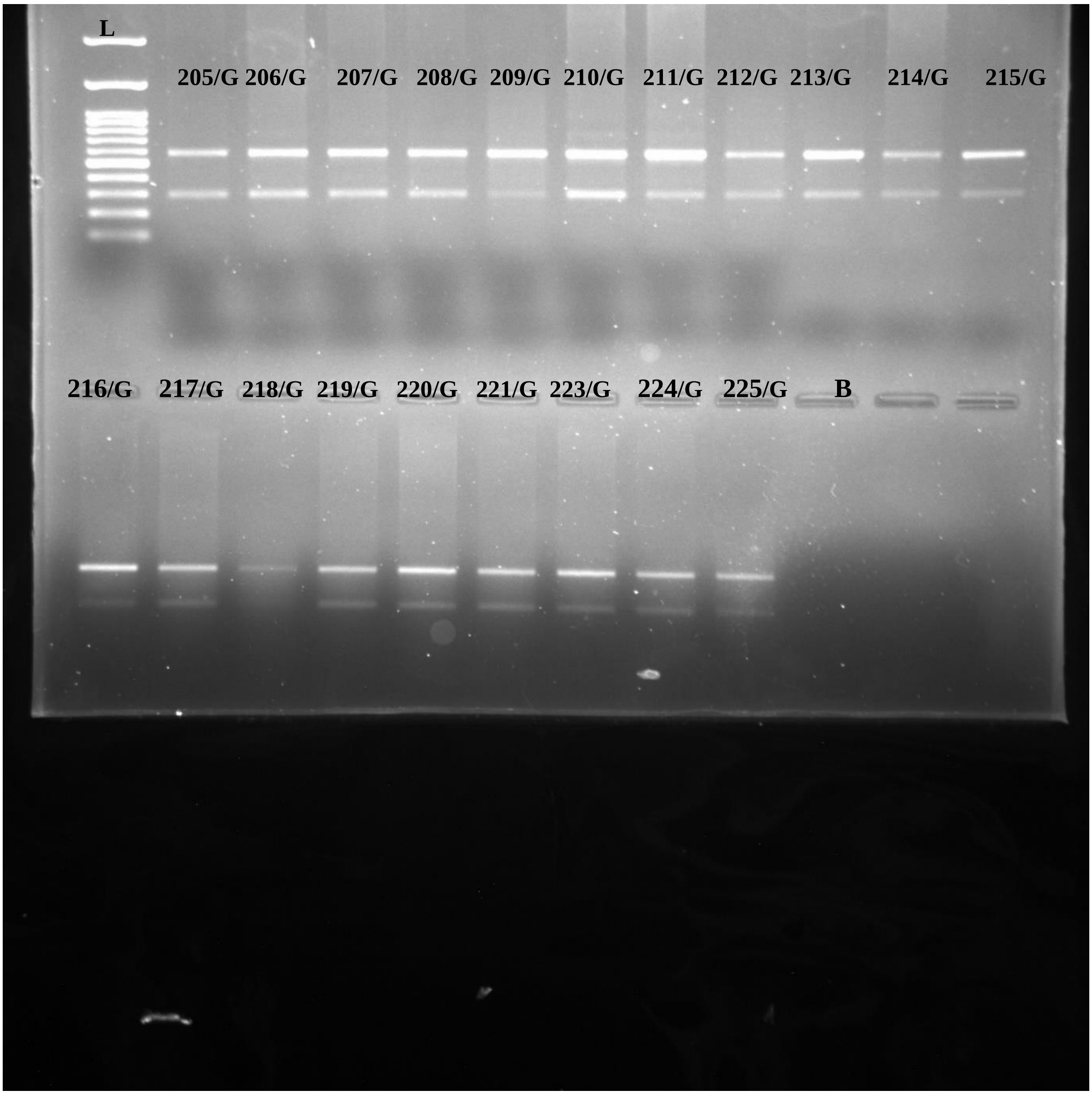
L
205/G 206/G 207/G 208/G 209/G 210/G 211/G 212/G 213/G 214/G 215/G
216/G 217/G 218/G 219/G 220/G 221/G 223/G 224/G 225/G B

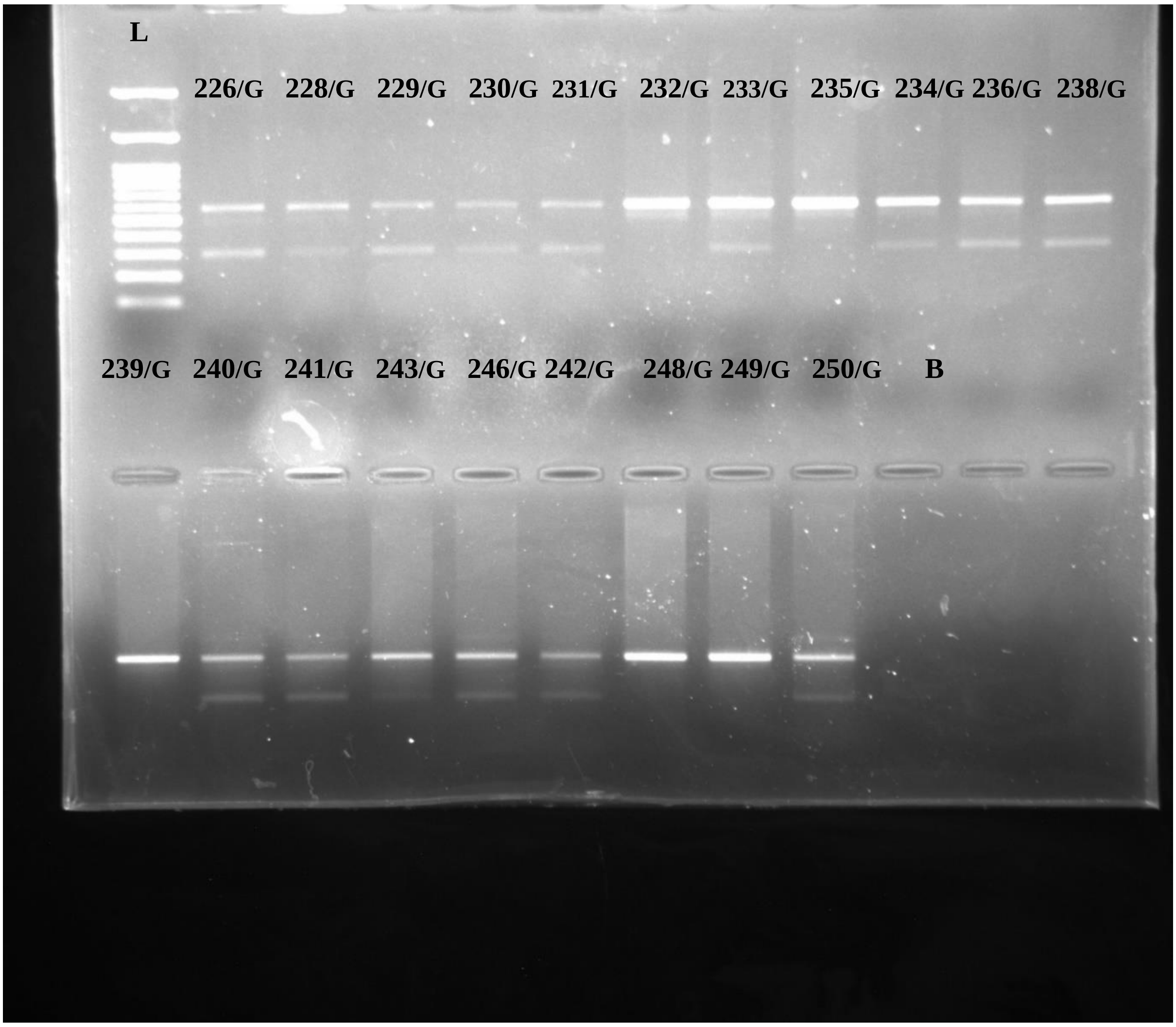
L
226/G 228/G 229/G 230/G 231/G 232/G 233/G 235/G 234/G 236/G 238/G
239/G 240/G 241/G 243/G 246/G 242/G 248/G 249/G 250/G B

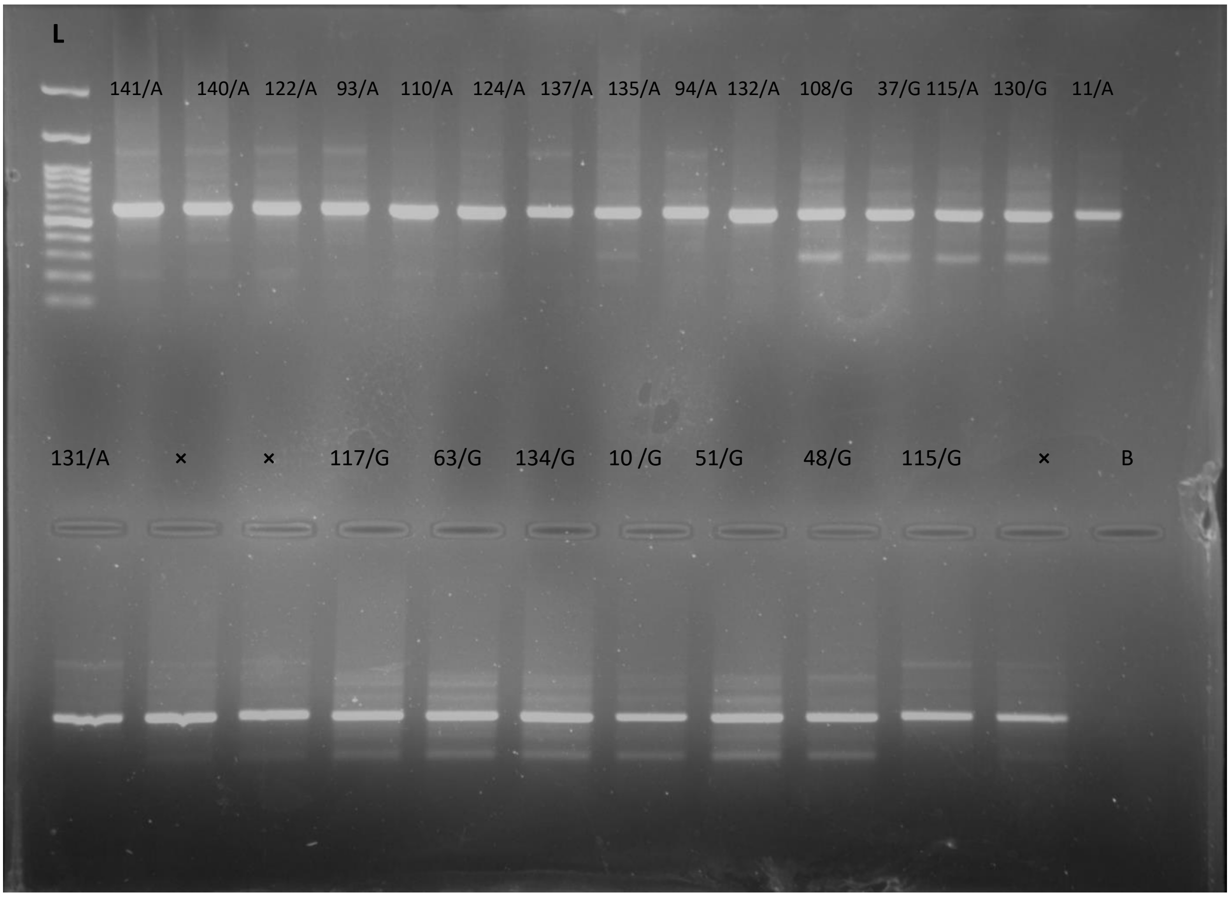
L
141/A
140/A
122/A
93/A
110/A
124/A
137/A
135/A
94/A
132/A
108/G
37/G
115/A
130/G
11/A
131/A
×
×
117/G
63/G
134/G
10 /G
51/G
48/G
115/G
×
B

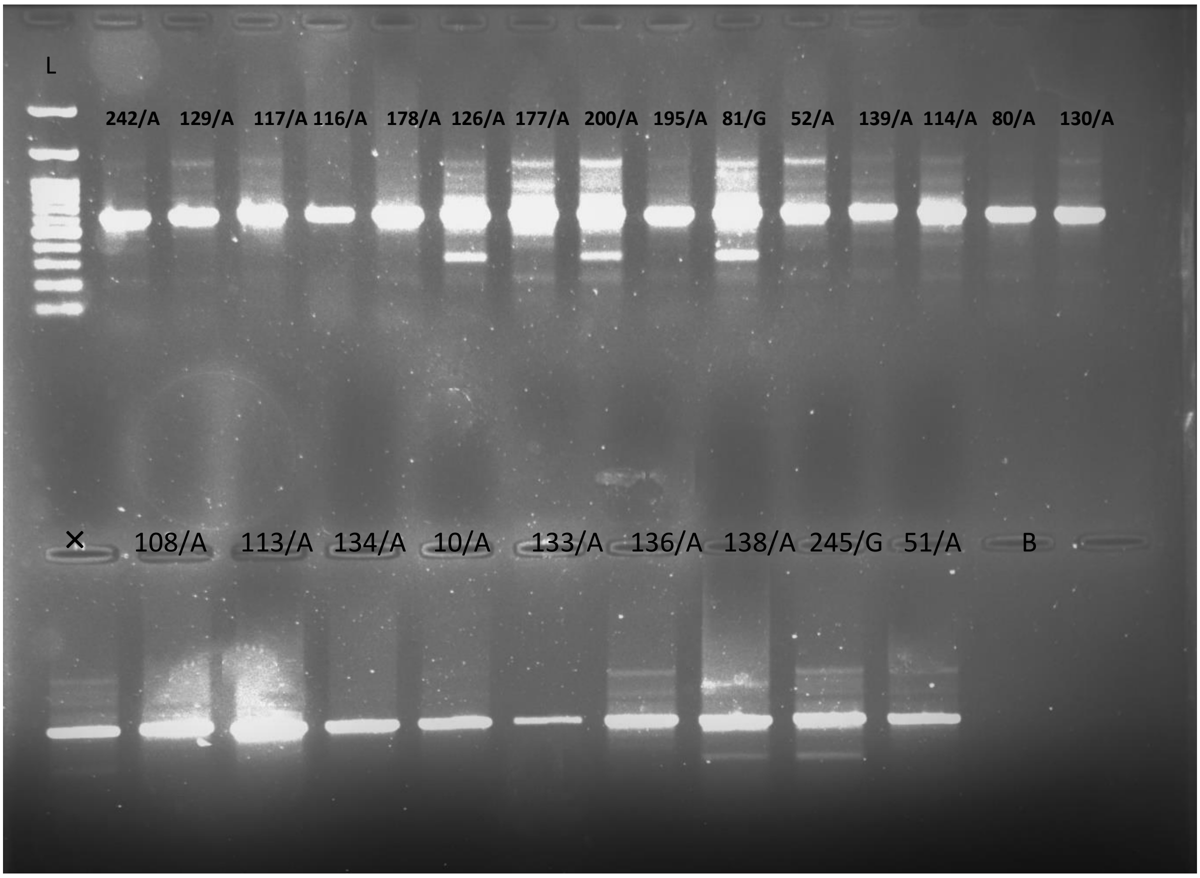
L
242/A 129/A 117/A 116/A 178/A 126/A 177/A 200/A 195/A 81/G 52/A 139/A 114/A 80/A 130/A
× 108/A 113/A 134/A 10/A 133/A 136/A 138/A 245/G 51/A B

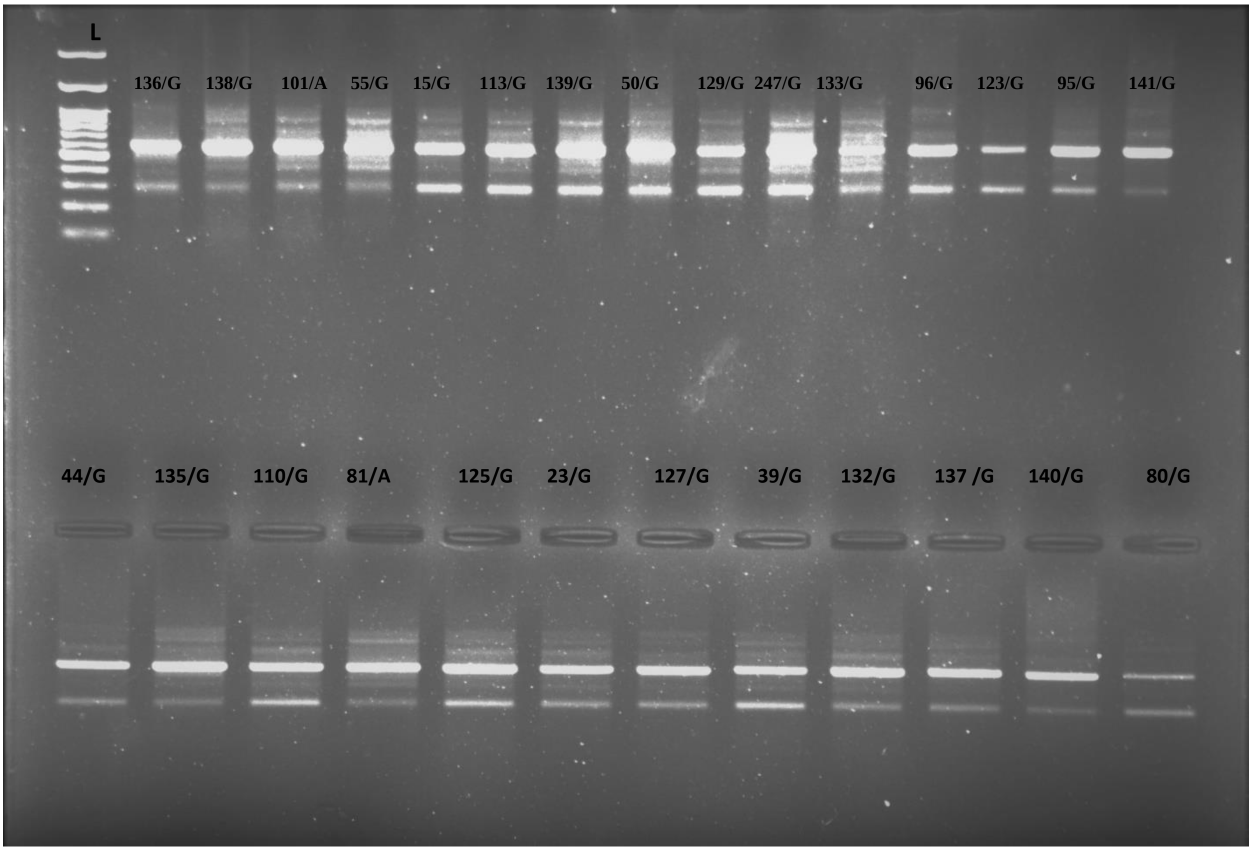
L
136/G 138/G 101/A 55/G 15/G 113/G 139/G 50/G 129/G 247/G 133/G 96/G 123/G 95/G 141/G
44/G 135/G 110/G 81/A 125/G 23/G 127/G 39/G 132/G 137 /G 140/G 80/G

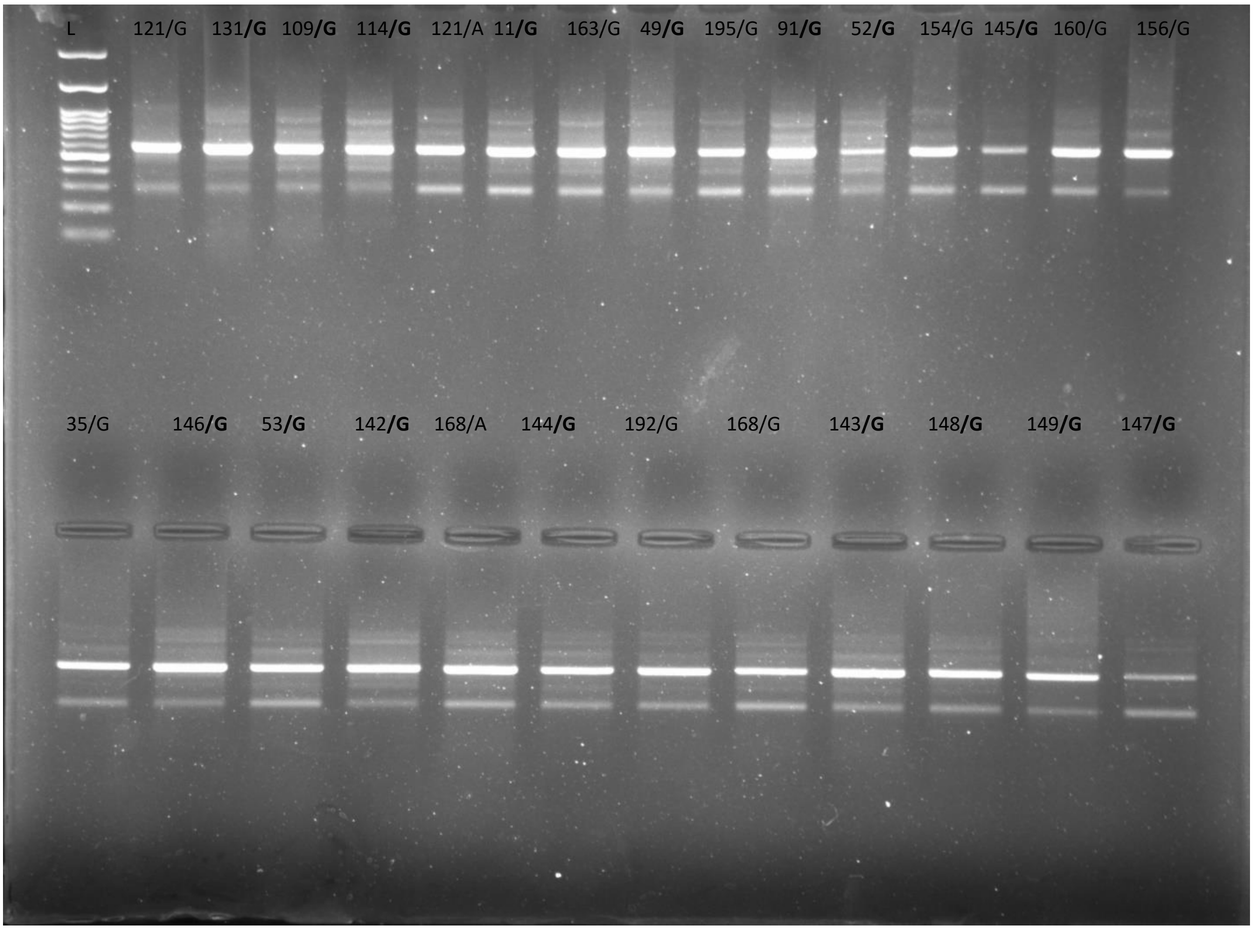
L 121/G 131/G 109/G 114/G 121/A 11/G 163/G 49/G 195/G 91/G 52/G 154/G 145/G 160/G 156/G
35/G 146/G 53/G 142/G 168/A 144/G 192/G 168/G 143/G 148/G 149/G 147/G

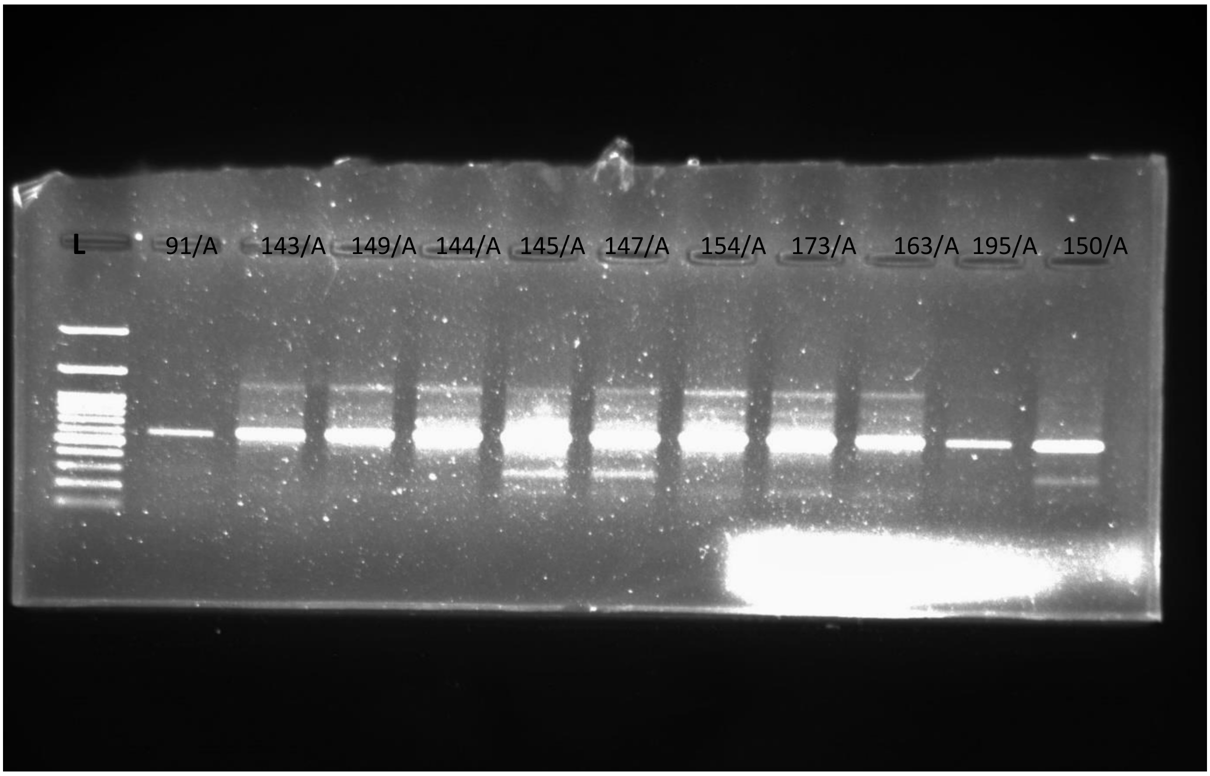
L 91/A 143/A 149/A 144/A 145/A 147/A 154/A 173/A 163/A 195/A 150/A

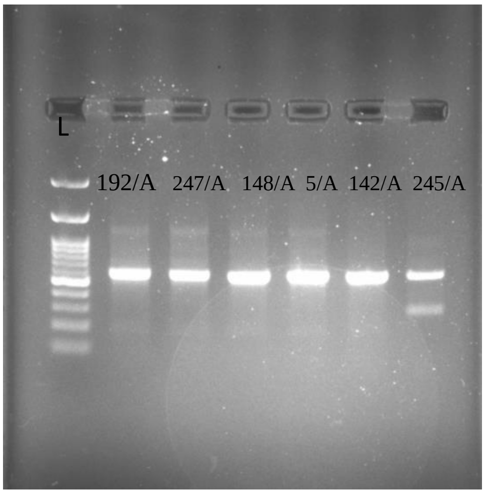
L
192/A
247/A
148/A
5/A
142/A
245/A

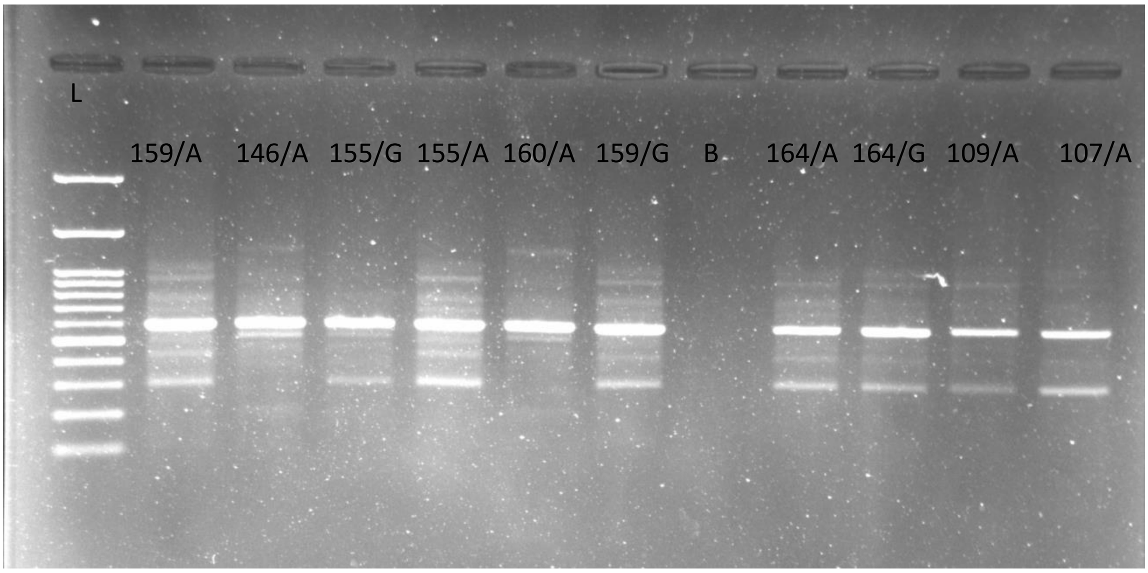
L
159/A
146/A
155/G
155/A
160/A
159/G
B
164/A
164/G
109/A
107/A

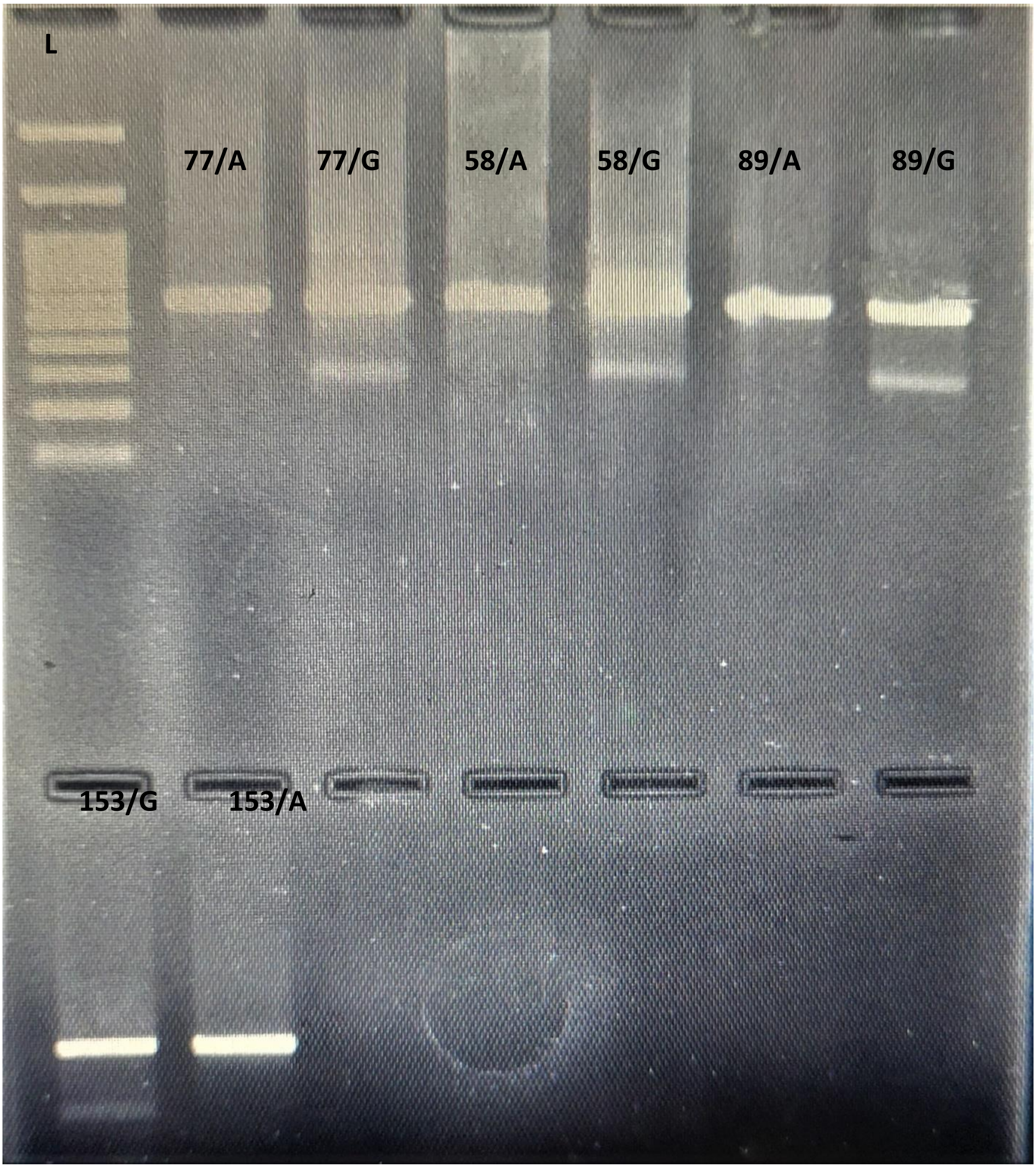
L
77/A
77/G
58/A
58/G
89/A
89/G
153/G
153/A

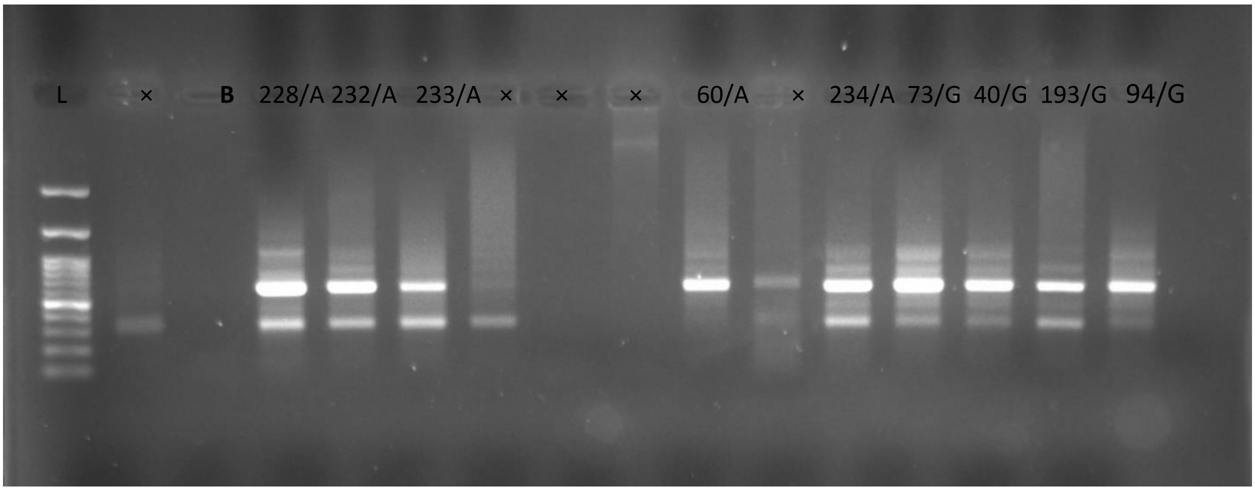
L × B 228/A 232/A 233/A × × × 60/A × 234/A 73/G 40/G 193/G 94/G

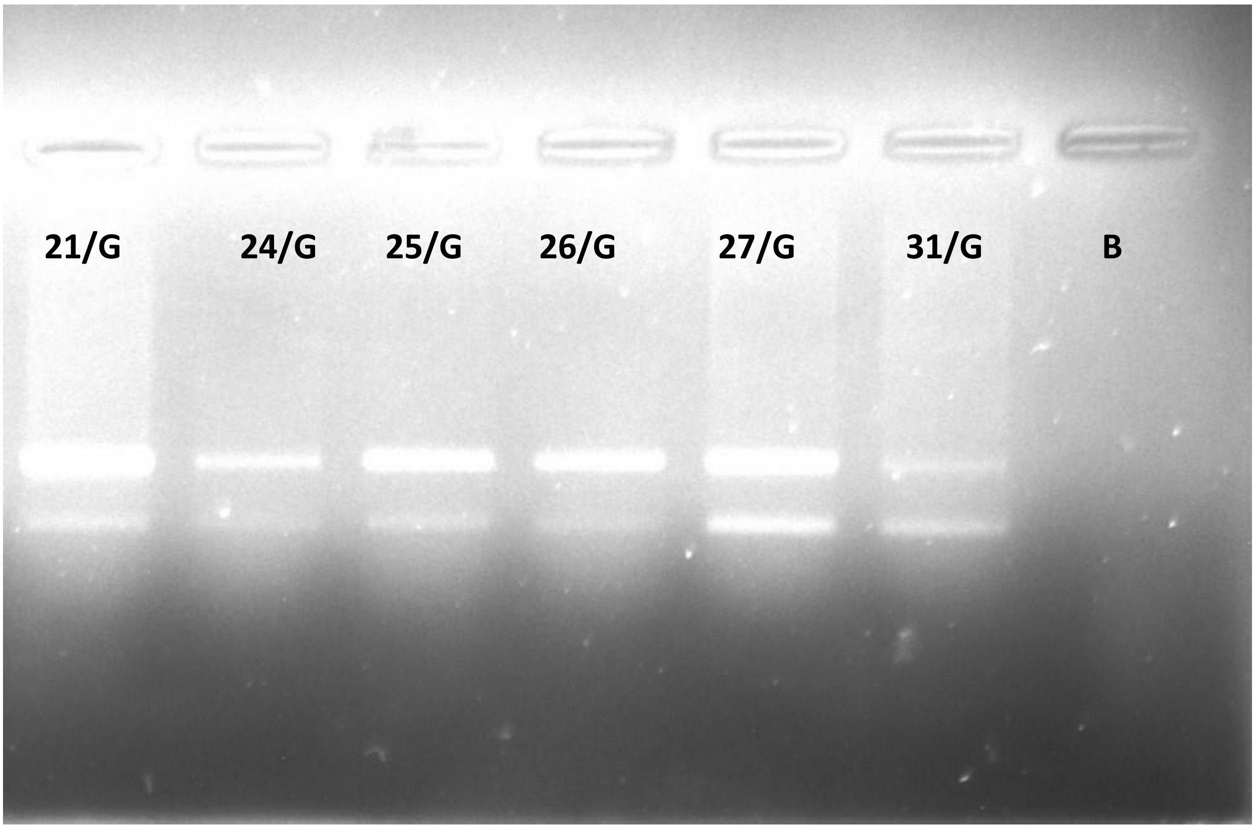
21/G 24/G 25/G 26/G 27/G 31/G B

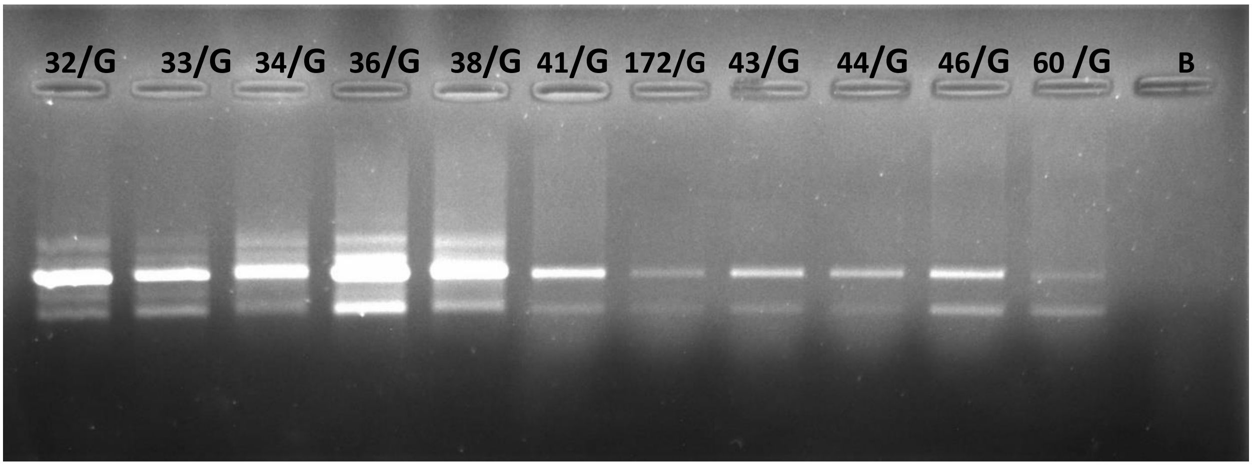
32/G 33/G 34/G 36/G 38/G 41/G 172/G 43/G 44/G 46/G 60 /G B

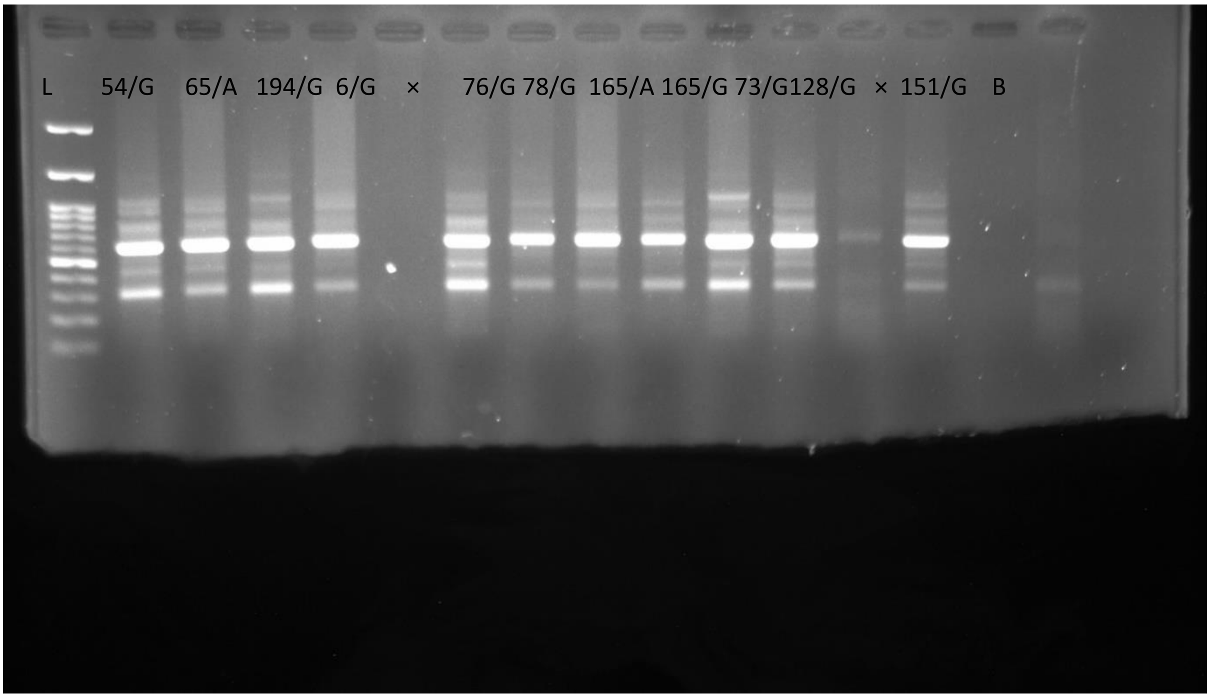
L 54/G 65/A 194/G 6/G × 76/G 78/G 165/A 165/G 73/G128/G × 151/G B

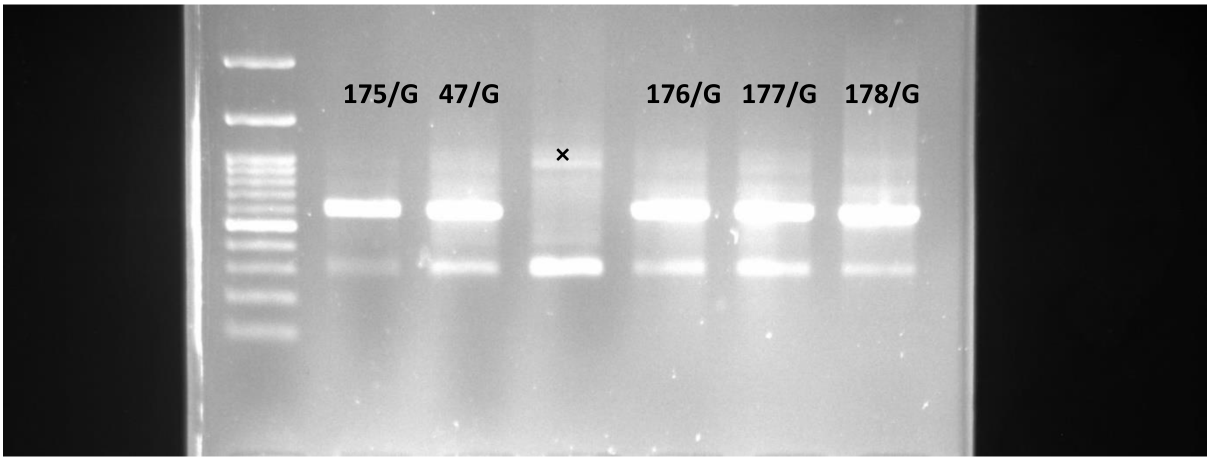
175/G 47/G 176/G 177/G 178/G
×

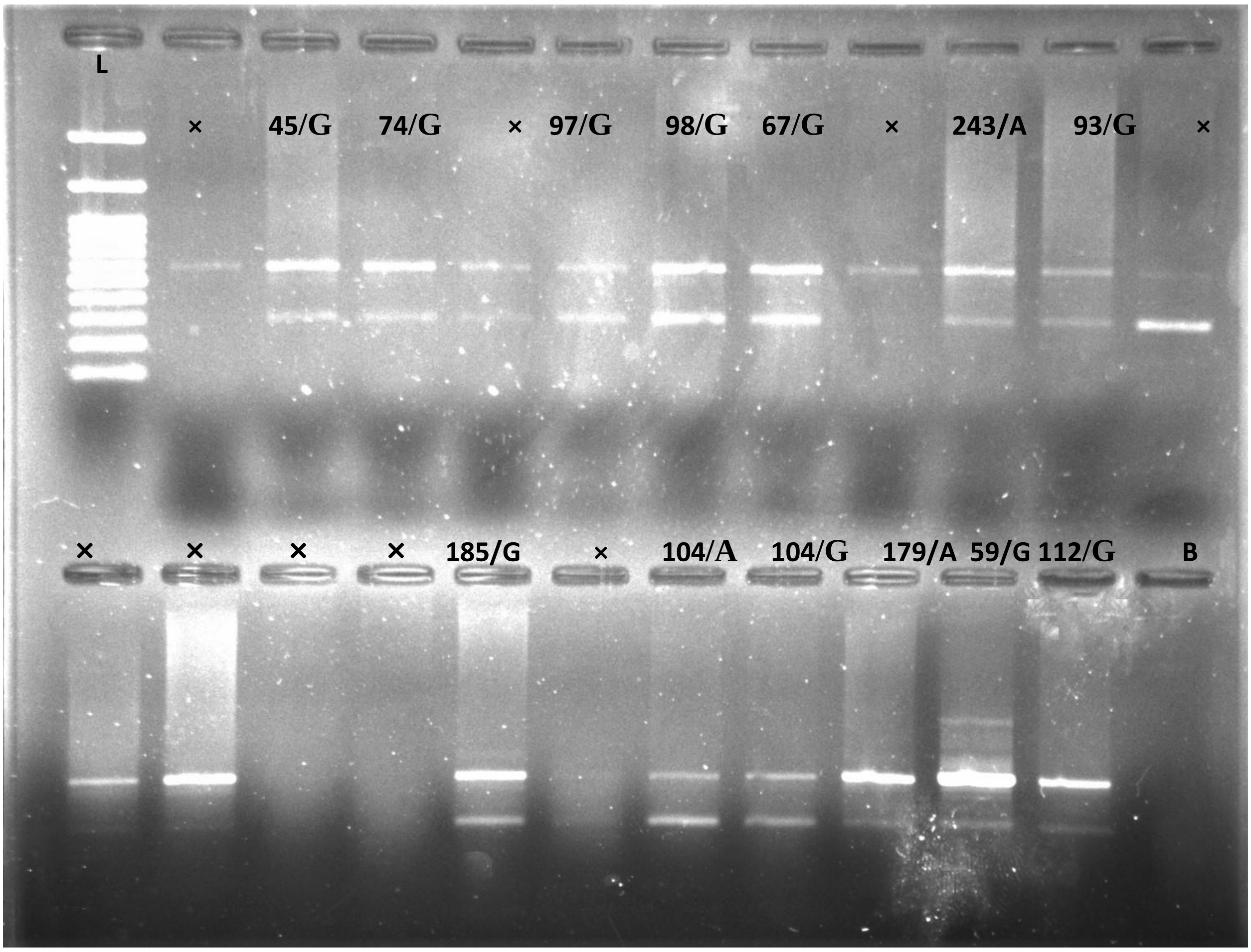
L
× 45/G 74/G × 97/G 98/G 67/G × 243/A 93/G ×
× × × × 185/G × 104/A 104/G 179/A 59/G 112/G B

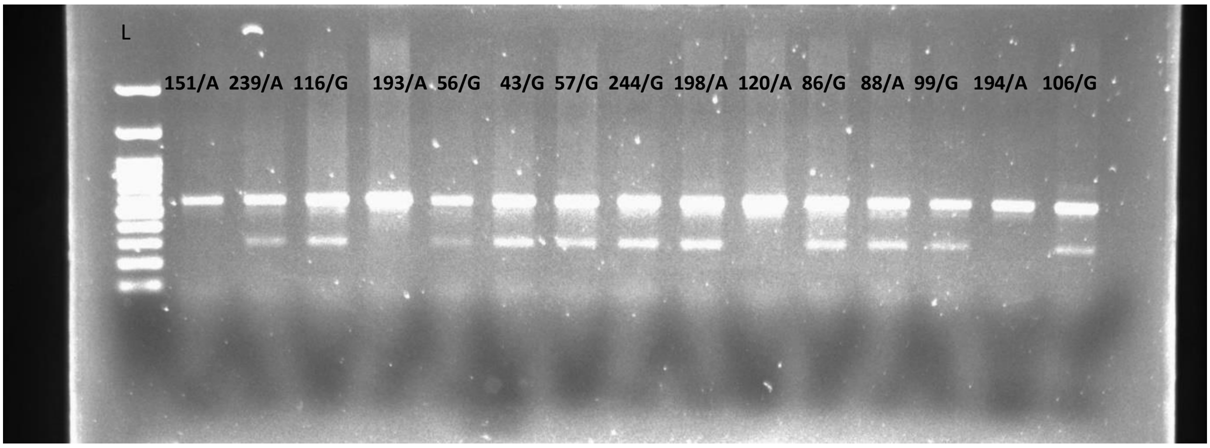
L
151/A 239/A 116/G 193/A 56/G 43/G 57/G 244/G 198/A 120/A 86/G 88/A 99/G 194/A 106/G

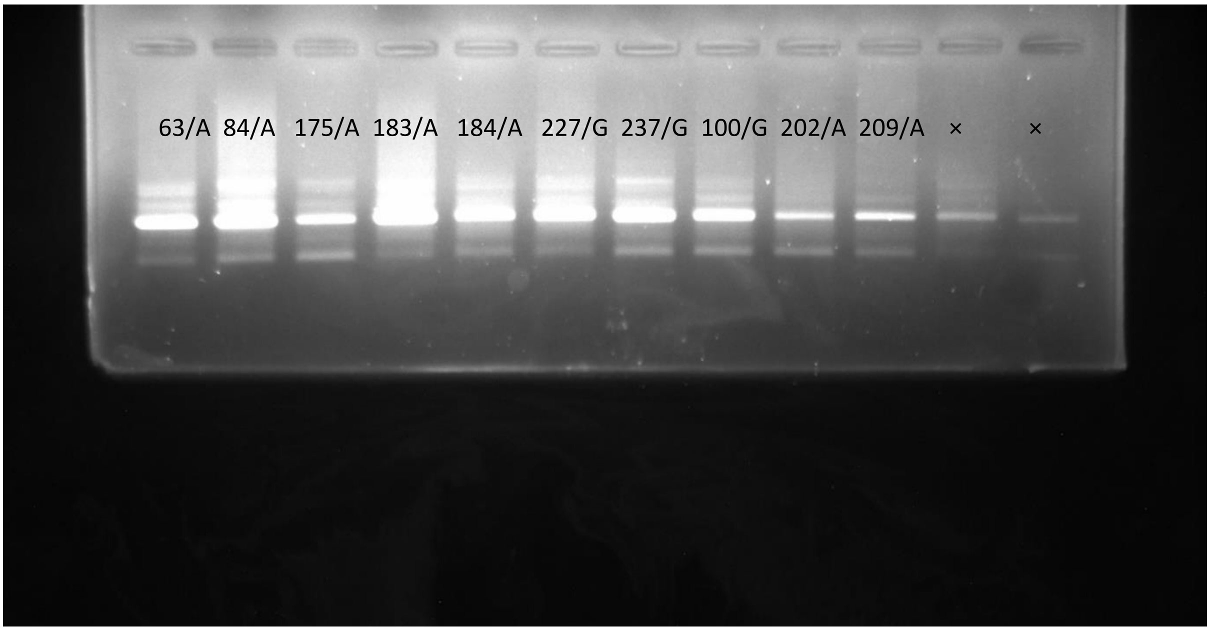
63/A 84/A 175/A 183/A 184/A 227/G 237/G 100/G 202/A 209/A × ×

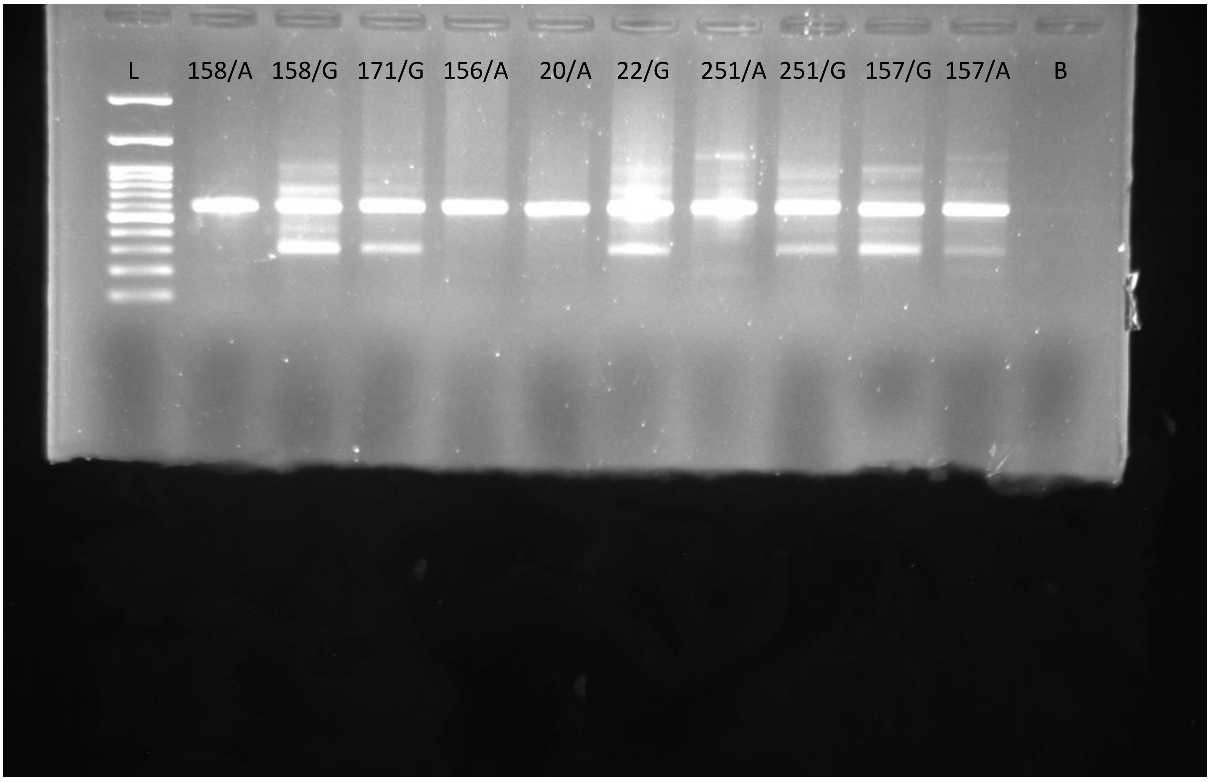
L 158/A 158/G 171/G 156/A 20/A 22/G 251/A 251/G 157/G 157/A B

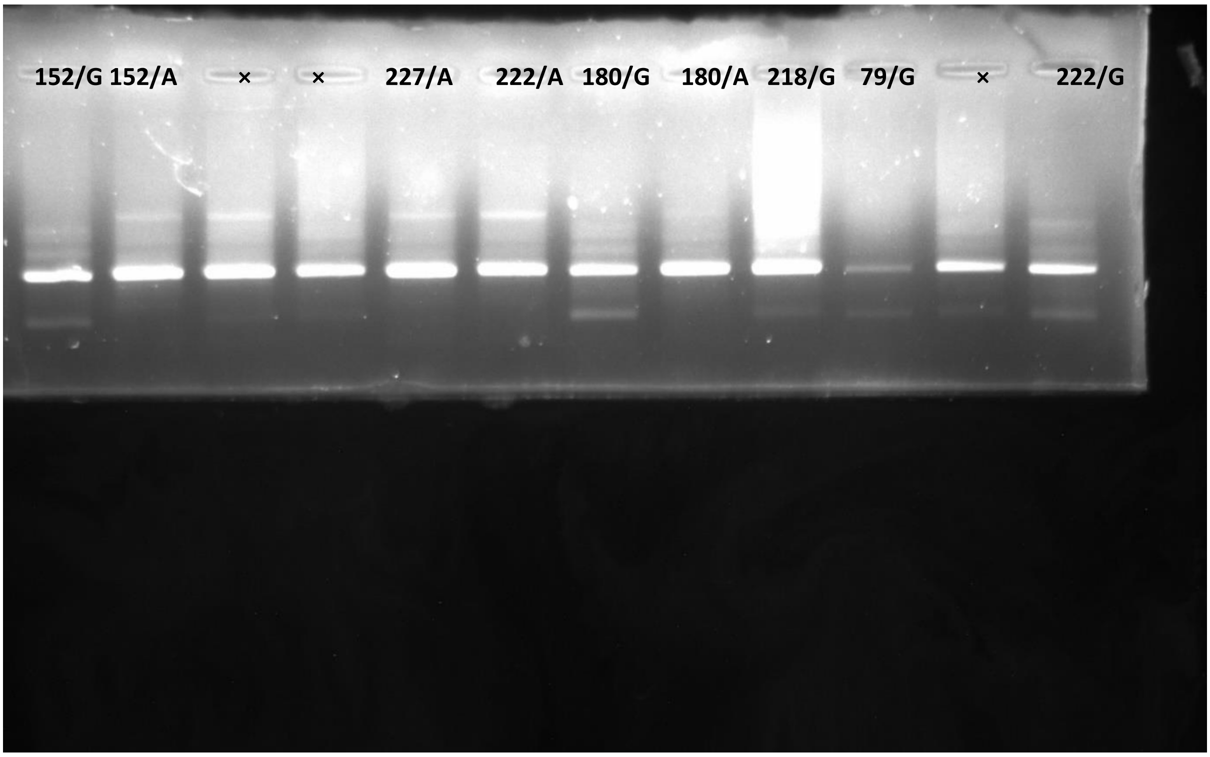
152/G 152/A × × 227/A 222/A 180/G 180/A 218/G 79/G × 222/G

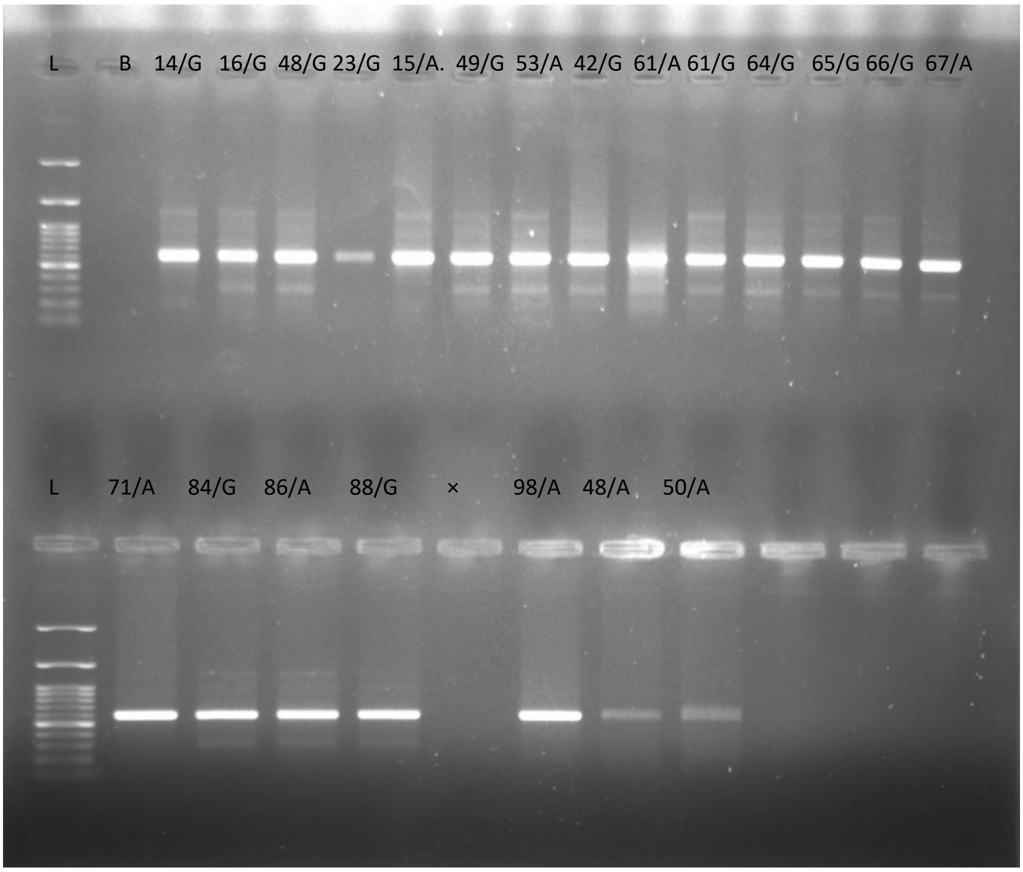
L B 14/G 16/G 48/G 23/G 15/A. 49/G 53/A 42/G 61/A 61/G 64/G 65/G 66/G 67/A
L 71/A 84/G 86/A 88/G × 98/A 48/A 50/A
